# Supplementary material for: The systematic relationships and biogeographic history of ornithischian dinosaurs
Source: PeerJ. 2015 Dec 22;3:e1523. doi: 10.7717/peerj.1523 (PMC4690359; doi:10.7717/peerj.1523)
Supplement: Table S1 — See Table S2 for character citations. [file peerj-03-1523-s001.docx]

Supplementary Table 1 **Description of characters used in the phylogenetic analysis of basal ornithischian relationships.** See Supplementary Table 2 for character citations.

1. Preorbital skull length less than or equal to 50% total skull length (0), greater than 50% (1).
2. Predentary bone absent (0), present (1).
3. Rostral bone absent (0), present (1).
4. Oral margin of the premaxilla smooth (0), denticulate (1).
5. Lateral surface of the oral margins of the premaxillae flat (0), everted (1).
6. Ventral margin of the premaxillalevel with the maxillary tooth row (0), ventrally deflected (1).
7. Anterior-most premaxillary tooth positioned at the anterior margin of the premaxilla (0), inset at least the width of one tooth crown (1).
8. Diastema between premaxilla and maxilla absent (0), present (1).
9. Diastema between premaxilla and maxilla flat (0), arched (1).
10. Anterior premaxillary foramen absent (0), present (1).
11. Premaxillary border of internal nares present (0), absent (1).
12. Anterodorsal surface of the premaxilla smooth (0), highly rugose (1).
13. Premaxillary posterolateral process does not exclude maxilla from nasal margin (0), does exclude maxilla from nasal margin (1).
14. Concavity within the posterior end of the premaxilla, near lateral margin, for receipt of the anterolateral boss of the maxilla absent (0), present (1).
15. Overlap of the dorsal process of the premaxilla onto the rostral process of the nasal absent (0), present (1).
16. Contact between premaxilla and lacrimal absent (0), present (1).
17. Premaxillary sulcus on the anterior process of the maxilla absent (0), present (1).
18. ‘Special foramina’ medial to dentary and maxillary tooth rowsabsent (0), present (1).
19. Buccal emargination on the maxilla absent (0), present (1).
20. Buccal emargination of maxilla consists of a gradual and shallow beveling of the ventrolateral surface of the maxilla (0), consists of a prominent ridge on the lateral surface of the maxilla (1).
21. Notch in maxilla for the lacrimal absent (0), present (1).
22. Fossa situated low along the boundary between the premaxilla and the maxilla absent (0), present (1).
23. Supraorbital absent (0), one supraorbital present (1), two or more supraorbitals present (2).
24. Supraorbital free and projects into orbit from contact with lacrimal and prefrontal (0), supraorbital incorporated into the orbital margin(1).
25. Supraorbital(s) extend across at least 71% of the maximum anteroposterior length of the orbit (0), 70% or less (1).
26. Greatest posterior expanse of the jugal greater than 25% height of skull (0), less than 25% (1).
27. Anterior process of the jugal straight (0), curved (1).
28. Anterior process of jugal situated dorsal to the maxilla (0), anterior process of jugal inserts into the maxilla (1).
29. Jugal barely contacts lacrimal (0), jugal touches lacrimal with more contact (1), butt joint between jugal and lacrimal (2).
30. Jugal contributes to the antorbital fenestra (0), does not reach antorbital fenestra (1).
31. Contact between jugal and postorbital faces anteriorly (0), contact faces partially laterally (1), postorbital inserts into a socket in the jugal (2).
32. Jugal dorsoventrally deeper than mediolaterally broad (0), broader than deep (1).
33. Jugal forms part of the posterior margin the infratemporal fenestra (0),only forms the ventral margin (1).
34. The anteroventral corner of the infratemporal fenestra, formed by the jugal, consists of an oblique or right angle (0), anteroventral corner of the infratemporal fenestra consists of an acute angle (1).
35. Contact between the jugal and quadratojugal consists of a butt or high angle scarf joint (0), the jugal overlaps the lateral surface of the quadratojugal posterodorsally and the medial surface posteroventrally (1).
36. Jugal does not contact quadrate (0), jugal does contact quadrate (1).
37. Jugal or quadratojugal contacts the quadrate near or slightly above the distal end (0), contacts positioned well above the distal end (1).
38. No boss or ornamentation present on lateral surface of the jugal (0), lateral surface of jugal ornamented, but no boss present (1), presence of a low boss on the lateral surface of the jugal (2), presence of a tall, posteriorly projecting boss on the lateral surface of the jugal (3).
39. Maxillary process on the medial side of the jugal is medially projected and modestly arched (0), maxillary process on the medial side of the jugal is straight and grooved (1), maxillary process on the medial side of the jugal is anteromedially projected and arched (2).
40. Ectopterygoid facet on the medial surface of the jugal consists of an abbreviated, deep groove (0), ectopterygoid facet consists of a rounded scar (1).
41. Quadratojugal foramen absent (0), present (1).
42. Dorsal process of the quadratojugal contacts the descending process of the squamosal (0), dorsal process of the quadratojugal does not contact the descending process of the squamosal (1).
43. Quadratojugal anteroposteriorly long and dorsoventrally short (0), quadratojugal anteroposteriorly short and dorsoventrally tall (1).
44. Quadratojugal contacts the quadrate along greater than 50% the total length of the quadrate (0), quadratojugal contacts the quadrate along less than 50% the total length of quadrate (1).
45. Proximal head of the quadrate recurved posteriorly (0), proximal head of quadrate straight (1).
46. Ventral portion of the quadrate shaft oriented vertically or anteroventrally angled (0), ventral portion of the quadrate shaft posteroventrally angled (1).
47. Body of the quadrate leans posteriorly (0), body of quadrate oriented vertically (1), body of quadrate leans anteriorly (2).
48. Jugal wing of the quadrate moderately developed (0), jugal wing of the quadrate shortened (1).
49. Ventral extent of the jugal wing of the quadrate positioned at or near the distal end of the quadrate (0), ventral extent of the jugal wing of the quadrate positioned above the distal end of the quadrate (1).
50. Pit in lateral side of quadrate at the base of the jugal wing present (0), absent (1).
51. Quadrate notch (tiny foramen between jugal wing of quadrate and quadratojugal) absent (0), present (1).
52. Distal condyles of the quadrate dorsomedially sloped or horizontally oriented (0), distal condyles of the quadrate dorsolaterally sloped (1).
53. The pterygoid wing of the quadrate arises at the dorsal head of the quadrate (0), pterygoid wing of the quadrate arises below the dorsal head of the quadrate (1).
54. Pterygoid wing of quadrate consists of a large, anteromedially extending fan of bone (0), pterygoid wing of the quadrate small (1).
55. Groove on the base of the posterior side of the pterygoid wing of the quadrate absent (0), present (1).
56. Length of ventral process of the squamosal less than 30% the total length of the quadrate (0), length of the ventral process of the squamosal greater than 30% the total length of the quadrate (1).
57. Paroccipital process oriented horizontally and slightly widened distally (0), distal end of paroccipital process ‘pendant-shaped ‘(1).
58. Parietosquamosal shelf absent (0), present (1).
59. Orbital edge of postorbital forms a smooth, continuous arc (0), orbital edge of postorbital possesses an anteriorly directed inflation into orbit (1).
60. Orbital margin of postorbital smooth (0), orbital margin striated and rugose (1).
61. Postorbital non-robust (0), postorbital robust (1).
62. Synovial socket for the head of the laterosphenoid positioned along the frontal-postorbital contact (0), synovial socket for the head of the laterosphenoid positioned only in the postorbital (1), synovial socket for the head of the laterosphenoid positioned only in the frontal (2), no synovial joint for the laterosphenoid present (3).
63. Greater than 25% of the frontal length participates in the orbital margin (0), less than 25% of the frontal length participates in the orbital margin.
64. Frontals extend over the entire orbit (0), frontals positioned only over the posterior half of orbit (1).
65. Frontals arched over orbit (0), frontals dorsally flattened (1).
66. Combined width of frontals greater than the total length of the frontals (0), combined width of the frontals less than the total length of the frontals (1).
67. Total length of frontals between 120% and 60% the total length of the nasals (0), total length of frontal less than 60% the total length of the nasals (1).
68. Length of the oral margin of the predentary less than the length of the oral margin of the premaxilla (0), length of the oral margin of the predentary equal to or greater than the length of the oral margin of the premaxilla (1).
69. Anterior tip of predentary pointed (0), anterior tip rounded (1).
70. Oral margin of the predentary smooth (0), oral margin denticulate (1).
71. Ventral process of the predentary present (0), ventral process of the predentary very reduced or absent (1).
72. One ventral process of the predentary (0), two ventral processes of the predentary (1).
73. Dentary symphysis ‘v-shaped’ (0), dentary symphysis ‘spout-shaped’ (1).
74. Anterior-most tip of dentary positioned within the dorsal 1/3 of the dentary (0), anterior-most tip positioned near mid-height of the dentary (1), anterior-most tip positioned within the lower 1/3 of the dentary (2), anterior-most tip curves ventrally below ventral margin of the dentary (3), anterior-most tip anterodorsally curved and positioned higher than the base of the dentary tooth row (4).
75. Dorsal and ventral margins of the dentary converge rostrally (0), dorsal and ventral margins of the dentary parallel (1).
76. Medial surface of the dentary straight (0), medial surface of the dentary medially arched (1).
77. Dentary depth just anterior to the rising coronoid process 20% or less the total length of the dentary (0), dentary depth 21% or more the total length of the dentary (1).
78. Dentary tooth row straight in lateral view (0), dentary tooth row sinuous in lateral view (1).
79. Coronoid process absent or weakly developed (0), coronoid process present (1).
80. Dentary does not contribute to the coronoid process (0), dentary does contribute to the coronoid process (1).
81. The posterior end of tooth row ends anterior to coronoid process (0), the posterior end of tooth the row is shrouded by the coronoid process in lateral view (1).
82. Coronoid process inconspicuous (0), coronoid process subtriangular (1), coronoid process subrectangular (2), coronoid process dorsally elongated with a lobe-shaped distal expansion (3).
83. The length of the mandible posterior to the coronoid process is 36% or greater the total length of mandible (0), the post-coronoid length of the mandible is between 25 and 35% the total length of the mandible (1), the post-coronoid length of the mandible is less than 25% the total length of the mandible (2).
84. Dorsal margin of the surangular convex or diagonal (0), dorsal margin of the surangular concave (1).
85. Surangular foramen absent (0), present (1).
86. Ridge or process on lateral surface of surangular, anterior to the jaw suture absent (0), a strong, anteroposteriorly extending ridge present (1), a dorsally directed, finger-like process present (2).
87. Distal condyles of the quadrate subequal (0), medial distal condyle larger (1), lateral distal condyle larger (2).
88. Maximum length of external nares less than 15% basal skull length (0), maximum length of external nares greater than 15% basal skull length (1).
89. External nares positioned close to the buccal margin and below the level of the orbit (0), external nares positioned higher than the maxilla (1).
90. Antorbital fenestra present (0), absent (1).
91. Antorbital fossa rounds smoothly onto maxilla along some part of its margin (0), antorbital fossa sharply defined or extended as a secondary lateral wall enclosing the fossa (1).
92. Maxillary fenestra absent (0), present (1).
93. Antorbital fossa triangular (0), antorbital fossa ovate or circular (1).
94. External opening of the antorbital fossa present and greater than 10% basal skull length (0), external opening of the antorbital fossa present and less than 10% basal skull length (1), external opening of the antorbital fossa absent (2).
95. Lower margin of the orbit circular (0), lower margin of the orbit subrectangular (1).
96. Ventral edge of the infratemporal fenestra extends to or below the ventral margin of the orbit (0), ventral edge of the infratemporal fenestra positioned well above the ventral margin of the orbit (1).
97. External mandibular fenestra present (0), absent (1).
98. Angle between the base and long axis of the braincase greater than 35 degrees (0), angle less than 35 degrees (1).
99. Median ridge on floor of the braincase absent (0), present (1).
100. Basioccipital and exoccipital contribute to the occipital condyle (0), exoccipital excluded from occipital condyle (1).
101. Nuchal crest on the supraoccipital absent (0), present (1).
102. Supraoccipital contributes to greater than 5% of the margin of the foramen magnum (0), supraoccipital contributes to less than 5% of the margin of the foramen magnum (1), supraoccipital excluded from the margin of the foramen magnum (2).
103. Posttemporal foramen positioned at the boundary between the parietals and the paroccipital process (0), posttemporal foramen positioned entirely within the opisthotic (1), posttemporal foramen positioned entirely within the squamosal (2).
104. Posttemporal foramen consists of an enclosed foramen (0), posttemporal foramen consists of a dorsally open groove (1).
105. Ventral keel on basioccipital absent (0), present (1).
106. Floor of basioccipitalflat (0), arched (1).
107. Basioccipital tubera lower than basisphenoid (0), basioccipital tubera level with basisphenoid (1).
108. Foramen magnum occupies over 30% of the dorsal margin of the occipital condyle (0), foramen magnum occupies between 30 and 20% of the dorsal margin of the occipital condyle (1), foramen magnum occupies less than 20% of the dorsal margin of the occipital condyle (2).
109. Length of basisphenoid (from base of the parasphenoid process to the posterior edge of the basisphenoid) less than the length of basioccipital (0), basisphenoid and basioccipital subequal in length (1), length of basisphenoid greater than the length of the basioccipital (2).
110. Foramen for cranial nerve V notches anteroventral edge of prootic (0), foramen for cranial nerve V completely enclosed within the prootic (1).
111. Premaxillary teeth present (0), absent (1).
112. Six teeth present in each premaxilla (0), between four and five teeth present in each premaxilla (1), one or less teeth present in each premaxilla (2).
113. Enlarged anterior canine tooth in dentary absent (0), present (1).
114. Ridges absent on dentary teeth, only simple denticles present (0), at least some denticles confluent with ridges that extend to base of crown on dentary teeth (1).
115. Apex of the maxillary teeth centrally placed (0), apex of maxillary teeth placed posterior of center (1).
116. Premaxillary teeth recurved, transversely flattened, constricted at the base (0), premaxillary teeth straight, subcylindrical, and unconstructed at the base. (1).
117. Space present between the roots and crowns of adjacent maxillary teeth (0), lack of space between crowns of adjacent maxillary teeth up through the occlusional margin (1), lack of space between roots and crowns of adjacent maxillary teeth (2), no space between crowns within each tooth position within the maxilla (3).
118. Apical ridge on the dentary teeth placed centrally or anterior of center (0), apical ridge of the dentary teeth placed posterior of center(1).
119. Maxillary tooth roots straight in anterior or posterior view (0), maxillary tooth roots curved in anterior or posterior view (1).
120. Distinct neck present below the crown of the maxillary teeth (0), maxillary crown tapers to root (1).
121. Maxillary teeth independently occlude (0), maxillary teeth share a continuous occlusional surface (1).
122. Lingual surface of maxillary teeth concave (0), lingual surface convex (1), lingual surface flat (2).
123. Distribution of enamel on maxillary and dentary teeth roughly equal on both sides (0), enamel primarily restricted to one side on maxillary and dentary teeth (1).
124. Ridges present on both sides of dentary crowns (0), ridges limited to one side of dentary crowns (1).
125. Maxillary crowns low and spade-like, rectangular, or triangular (0), maxillary crowns high and diamond-shaped (1), maxillary crowns laterally flattened and posteriorly recurved (2), maxillary crowns conical (3).
126. Twelve or less teeth present in each maxilla (0), between thirteen and nineteen teeth present in each maxilla (1), twenty or more teeth present in each maxilla (2).
127. Maxillary teeth possess a smooth face with simple denticles (0), maxillary teeth possess ridges confluent with denticles that extend to base of crown (1).
128. All ridges on maxillary teeth equally prominent (0), one ridge on maxillary teeth more prominent than the rest (1).
129. Cingulum on maxillary teethpresent (0), cingulum on maxillary teeth absent (1).
130. Cingulum on dentary teeth absent (0), cingulum on dentary teeth present (1).
131. Maxillary teeth positioned near the lateral margin (0), maxillary teeth inset medially (1).
132. Maxillary crown height less than 50% length (0), maxillary crown height between 50 and 90% crown length (1), maxillary crown height subequal to the crown length (2), maxillary crown height between 110 and 150% crown length (3), maxillary crown height greater than 150% crown length (4).
133. Dentary crowns less than 50% higher than mesiodistally wide (0), dentary crowns greater than 50% higher than mesiodistally wide (1).
134. Denticles extend beyond apical third of maxillary and dentary tooth crowns (0), denticles restricted to apical third of maxillary and dentary tooth crowns (1).
135. Dentary tooth roots straight in anterior or posterior view (0), dentary tooth roots curved in anterior or posterior view (1).
136. Dentary crowns rectangular, triangular, or leaf-shaped (0), dentary teeth lozenge-shaped (1), dentary teeth laterally flattened, posteriorly recurved (2), dentary teeth conical (3).
137. Thirteen or less dentary teeth present (0), between fourteen and seventeen dentary teeth present (1), eighteen or more dentary teeth present (2).
138. Fewer than ten ridges present on dentary teeth (0), ten or more ridges present on dentary teeth (1).
139. All ridges on the dentary teeth equally prominent (0), one ridge more prominent than the rest on the dentary teeth (1).
140. Anterior two dentary teeth similar in morphology to more posterior dentary teeth (0), anterior two dentary teeth lack denticles, first tooth strongly reduced (1).
141. Less than ten cervical vertebrae (0), ten or more cervical vertebrae (1).
142. Cervical vertebrae plateocoelous or amphicoelous (0), opisthocoelous (1).
143. Ventral surface of the cervical vertebrae rounded (0), presence of a broad, flattened keel on the ventral surface of the cervical vertebrae (1), presence of a sharp ventral keel on the ventral surface of the cervical vertebrae (2).
144. Anterior cervical centra less than 1.5 times longer than tall (0), length of anterior cervical centra equal or greater than 1.5 times longer than tall (1).
145. Epipophyses on anterior cervical three present (0), absent (1).
146. Dorsal neural spines arise anteriorly or are centered over centrum (0), dorsal neural spines posteriorly positioned on centrum (1).
147. Fourteen or less dorsal vertebrae present (0), fifteen dorsal vertebrae present (1), sixteen dorsal vertebrae present (2), seventeen or more dorsal vertebrae present (3).
148. Sacrum composed of three or fewer fused vertebral centra (0), sacrum composed of between four and five fused vertebral centra (1), sacrum composed of six fused vertebral centra (2), sacrum composed of seven or more fused vertebra centra (3).
149. Sacral neural spines less than twice the height of the sacral centra (0), sacral neural spines between 2 and 2.5 times the height of the sacral centra (1), sacral neural spines greater than 2.5 times the height of the sacral centra (2).
150. Sacral neural spines lean posteriorly (0), sacral neural spines lean anteriorly (1).
151. Height of neural spine on proximal caudal vertebrae less than 1.5 times taller than the height of the centrum (0), height of neural spine greater than 1.5 times taller than the height of the centrum (1).
152. Caudal neural spines positioned entirely over their respective caudal centra (0), caudal neural spines extend beyond their own centrum (1).
153. Caudal ribs positioned entirely on caudal centra (0), caudal ribs positioned along neurocentral suture (1), caudal ribs positioned on neural arch (2).
154. First caudal vertebra bears the longest caudal rib (0), longest caudal rib positioned posterior to first caudal vertebra (1).
155. Axial epiphyses present at least vestigially (0), axial epiphyses absent (1).
156. Transition from tuberculum and capitulum of dorsal ribs from vertical to near horizontal occurs between dorsal vertebrae 2 and 4 (0), transition occurs between dorsal vertebrae 5 and 6 (1), transition occurs between dorsal vertebrae 6 and 8 (2).
157. Partial ossification of the sternal segments of the cranial dorsal ribs absent (0), present (1).
158. Scapular spine on the anterodorsal corner of the scapula low or broad (0), scapular spine sharp and pronounced (1).
159. Minimum thickness of scapular neck less than 20% maximum length of the scapula (0), minimum width of neck greater than 20% maximum length of the scapula (1).
160. Width of coracoid less than 60% the length of the coracoid (0), width of coracoids between 61-100% the length of the coracoid (1), width of the coracoid greater than the length of the coracoid (2).
161. Coracoid foramen enclosed within the coracoids (0), coracoid foramen open along coracoid-scapula articular contact surface (1).
162. Length of sternal process of the coracoids [measured from the tip of the sternal process of the coracoid to the base of the coracoid notch less than 70% the width of the sternal process [measured at the level of the base of the coracoids notch] (0), length of sternal process greater than 70% the width of the sternal process (1).
163. Ovoid fossa positioned anteroventral to the glenoid fossa on the coracoid absent (0), present (1).
164. Sternal plates crescent-shaped (0), sterna plates hatchet-shaped (1), sterna plates expanded along the anterior and posterior ends and constricted in the middle (2).
165. Humerus longer than or subequal to scapula (0), scapula longer than humerus (1).
166. Total forelimb length [measured from the head of the humerus to tip of the manus] longer than 40% the total length of the hind limb [measured from the head of the femur to tip of the pes] (0), total forelimb length equal to or less than 40% the total length of the hind limb (1).
167. Shaft of the humerusstraight (0), shaft of the humerus exhibits at least a modest caudal flexure at the level of the deltopectoral crest (1).
168. Deltopectoral crest rounded in lateral view (0), deltopectoral crest angular in lateral view (1), deltopectoral crest inconspicuous (2).
169. Olecranon process low (0), olecranon process moderately developed (1), olecranon process well-developed (2).
170. Cross-sectional shape of the ulna at midshafttriangular or oval (0), cross-sectional shape of the ulna at midshaft cylindrical (1).
171. Shaft of the ulna straight (0), shaft of the ulna bowed (1).
172. Minimal radial width less than 10% the length of the radius (0), minimal radial width greater than 10% the length of the radius (1).
173. Carpus unfused (0), carpus fused (1).
174. Metacarpal I greater than 50% the length of metacarpal ii (0), metacarpal I less than 50% the length of metacarpal ii (1).
175. Manual digit I oriented less than 25 degrees from digit iii (0), manual digit I oriented between 25 and 60 degrees from digit iii (1), manual digit I oriented at an angle greater than 60 degrees from digit iii (2).
176. Ungual of manual digit I claw-like (0), ungual subconical (1), ungual absent (2).
177. First phalanx of manual digits ii-iv less than twice the size of second phalanx (0), first phalanx greater than twice the size of second phalanx (1).
178. Unguals on manual digits ii and iii longer than wide (0), unguals on manual digits ii and iii wider than long (1).
179. Four phalanges present in manual digit iii (0), three phalanges present in manual digit iii (1).
180. Three phalanges present in manual digit IV (0), two phalanges present in manual digit IV (1), one phalanx present in manual digit iv (2).
181. Two phalanges present in manual digit V (0), one phalanx present in manual digit V (1), phalanges absent in manual digit V (2).
182. Acetabulum on ilium normal to high (0), acetabulum on ilium short to long (1).
183. Ventral acetabular flange on the ilium present (0), absent (1).
184. Supra-acetabular rim on the ilium weakly developed or absent (0), supra-acetabular rim on the ilium strongly developed (1).
185. Dorsal margin of the ilium straight to slightly convex in lateral view (0), dorsal margin of the ilium sinuous in lateral view (1).
186. External surface of the preacetabular process of the ilium laterally facing and roughly in the same plane as the body of the ilium (0), external surface of the preacetabular process of the ilium twisted about its long axis (1).
187. Anterior tip of the preacetabular process of the ilium situated posterior to the anterior tip of the pubic peduncle of the ilium (0), anterior tip of the preacetabular process of the ilium situated anterior to the anterior tip of the pubic peduncle of the ilium (1).
188. Length of the postacetabular process of the ilium between 40 and 21% the total length of the ilium (0), postacetabular process of the ilium less than 20% the total length of the ilium (1), postacetabular process of the ilium greater than 40% the total length of the ilium (2).
189. Brevis shelf on the ilium oriented vertically (0), brevis shelf on the ilium extends medially in a roughly horizontal plane (1).
190. Ischiac peduncle of the ilium is not supported by a sacral rib (0), ischiac peduncle of the ilium supported by a sacral rib (1).
191. Lateral swelling of the ischiac peduncle of the ilium absent (0), present (1).
192. Pubic peduncle of the ilium more robust than the ischial peduncle and expands in lateral view (0), pubic peduncle of the ilium tapers distally and is smaller than the ischial peduncle (1).
193. Pubis not secondarily supported (0), pubis supported only by sacral rib (1), pubis supported directly by at least one sacral centrum (2).
194. Pubis anteroventrally facing (0), pubis vertically oriented (1), pubis posteroventrally rotated (2).
195. Anterior process of the pubispresent and straight (0), anterior process of the pubis present and dorsally curved (1), anterior process of the pubis absent (2).
196. Angle between prepubic process and pubic shaft greater than 150 degrees (0), angle less than 100 degrees (1).
197. Prepubic process of the pubis short and peg-shaped (0), prepubic process of the pubis mediolaterally flattened(1), prepubic process of the pubis rod-shaped (2), prepubic process of the pubis dorsoventrally flattened (3).
198. Length of the prepubic process of the pubis (measured from the obturator notch) less than 20% the total length of the ilium (0), prepubic process of the pubis greater than 20% the total length of the ilium (1).
199. Iliac and pubic peduncles of the ischium continuous, but separated by a fossa (0), iliac and pubic peduncles distinct and separated by a concave surface (1).
200. Pubic peduncle of ischium larger than iliac peduncle (0), peduncles subequal or iliac peduncle larger than pubic peduncle (1).
201. Dorsal margin of ischial shaft straight at mid-length in lateral view (0), caudodorsally convex at mid-length in lateral view (1), caudodorsally concave at mid-length in lateral view (2).
202. Groove on the dorsal edge of the ischium absent (0), present (1).
203. Tab-shaped obturator process absent (0), present (1).
204. Obturator process placed within the proximal 40% of the ischium (0), obturator process placed within the distal 60%(1).
205. Ischial shaft flat and blade-like (0), ischial shaft ovoid to subcylindrical (1).
206. Ischial symphysis present along at least 50% of the ischial shaft (0), ischial symphysis only present distally (1).
207. Enlarged 'foot' on the distal end of the ischial shaft absent (0), present (1).
208. Minimum diameter of the femur less than 15% of total femur length (0), minimum diameter of the femur greater than 15% of total femur length (1).
209. Femoral shaft straight in anterior view (0), femoral shaft distinctly bowedin anterior view (1).
210. Angle between the neck of the femoral head and the femoral shaftless than or equal to 100 degrees (0), angle greater than 100 degrees (1).
211. Neck-like constriction under the head of the femur absent (0), present (1).
212. Trench between the greater trochanter and the head of the femur absent (0), present (1).
213. Lateral surface of the greater trochanter of femur convex (0), lateral surface of the greater trochanter flattened (1).
214. Intertrochanteric notch between the lesser and greater trochanters on the femur present (0), absent (1).
215. Dorsal margin of the lesser trochanter of the femur lower than or equal to the height of the greater trochanter (0), dorsal margin of lesser trochanter higher than the height of the greater trochanter (1).
216. Lesser trochanter of femur positioned anterior and medial to greater trochanter (0), lesser trochanter positioned anterior and somewhat lateral to greater trochanter (1).
217. Lesser trochanter of femur consists of a prominent crest (0), lesser trochanter similar in width to the greater trochanter and separated from it by a wide cleft (1), lesser trochanter narrow and closely appressed to the greater trochanter (2).
218. Dorsal margin of the lesser trochanter substantially lower than the head of the femur (0), dorsal margin of the lesser trochanter approximately the same height as the head of the femur (1).
219. Fourth trochanter of the femur ‘mound-like’ (0), fourth trochanter a sharp ridge (1), fourth trochanter ‘pendant-shaped’ (2), fourth trochanter subtriangular (3), fourth trochanter vestigial, consisting of a rugosity or scar (4).
220. Insertion scar of m. Caudifemoralis longus on femur extends from fourth trochanter onto medial surface of femoral shaft (0), insertion scar of m. Caudifemoralis longus widely separated from fourth trochanter and restricted to the medial surface of the femoral shaft (1).
221. Fourth trochanter entirely on the proximal half of the femur (0), fourth trochanter placed at or below midshaft of the femur (1).
222. Anterior intercondylar groove on the distal femur absent (0), present (1).
223. Anterior intercondylar groove of the femur broad, shallow, and 'v-shaped’ with the edges of the groove meeting at an angle of greater than 90 degrees (0), anterior intercondylar groove tight, deep, and 'v-shaped’ with edges of the groove meeting at less than a 90 degree angle (1), anterior intercondylar groove deep, narrow, 'u-shaped,’ and partially enclosed by a slight expansion of the medial condyle (2), anterior intercondylar groove 'u-shaped’ and partially enclosed by expansions of both distal condyles (3), anterior intercondylar groove consists of a canal fully enclosed by fusion of lateral and medial condyles (4).
224. Posterior intercondylar groove on the femurfully open (0), medial condyle inflated and at least partially covering the intercondylar groove (1).
225. Posterior intercondylar groove extends less than ¼ the length of the femur (0), posterior intercondylar groove extends greater than ¼ the length of the femur (1).
226. Femur shorter than or equal to tibia in length (0), femur longer than tibia (1).
227. Tibia triangular in transverse section (0), tibia rounded in transverse section (1).
228. Lateral proximal condyles on the tibia equal in size (0), fibular condyle smaller (1), only one lateral proximal condyle present (2), fibular condyle larger (3).
229. Lateral extension of the tibial posterior flange does not reach fibula (0), lateral extension of the tibial posterior flange extends posterior to the medial margin of the fibula (1), lateral extension of the tibial posterior flange extends posterior to entire distal end of the fibula and calcaneum (2), lateral extension of the tibial posterior flange absent (3).
230. Cnemial crest of tibiastraight (0), cnemial crest arcs anterolaterally (1).
231. Cnemial crest of tibia rounded (0), cnemial crest sharply defined (1).
232. Lateral distal condyle on the femur subequal to the medial distal condyle (0), lateral distal condyle 80-60% the size of the medial distal condyle (1), lateral distal condyle 59-50% the size of the medial distal condyle (2), lateral distal condyle 49-40% the size of the medial distal condyle (3), lateral distal condyle 39-30% the size of the medial distal condyle (4), lateral distal condyle 29-20% the size of the medial distal condyle (5).
233. Fibular shaft elliptical or rounded in transverse section at mid-length (0), fibular shaft ‘d-shaped’ in transverse section throughout its length (1).
234. Ascending process of astragalus short (0), ascending process triangular and ‘tooth-like’ (1), ascending process ‘spike-like’ (2), ascending process relatively large (3).
235. Posterior side of astragalus low (0), high (1).
236. Height of anterior side of astragalus high (0), extends moderately high (1), height of anterior side low (2).
237. Articular surface for fibula on the astragalus covers more than 30% of the proximal surface (0), articular surface covers less than 30% of proximal surface of astragalus (1).
238. Angle between the articular facets for the tibia and fibula on the calcaneum greater than 120 degrees (0), angle less than 120 degrees (1).
239. Three or more distal tarsals present (0), two or less distal tarsals present (1).
240. Medial distal tarsal blocky, thin, and rectangular in proximal view (0), medial distal tarsal round in proximal view (1).
241. Medial distal tarsal does not cover any part of the proximal surface of metatarsal II (0), medial distal tarsal covers at least a portion of the proximal surface of metatarsal II (1).
242. Lateral distal tarsal ‘square-shaped’ (0), ‘kidney-shaped’ (1).
243. Metatarsal I present (0), absent (1).
244. Metatarsal V present (0), absent (1).
245. Metatarsal V less than 25% the length of metatarsal III (0), between 25% and 50% (1), greater than 50% (2).
246. Diameter of the midshafts of metatarsals I and V subequal to or greater than metatarsals II-IV (0), diameter of the midshafts of metatarsals I and V less than metatarsals II-IV (1).
247. Four functional digits in the pes (0), three functional digits in the pes (1).
248. Phalanges in pedal digit I present (0), absent(1).
249. Unguals on pedal digits II-IV longer than wide (0), approximately as wide as or wider than long (1).
250. Ossified hypaxial tendons along the tail absent (0), present (1).
251. Epaxial ossified tendons absent (0), present (1).
252. Epaxial tendons longitudinally arranged into a single layer (0), arranged in a double-layered lattice (1).
253. Postcranial osteoderms absent (0), present (1).
254. Dermal sculpturing of the skull and/or mandible absent (0), present (1).
255. Premaxillae unfused (0), fused (1).
