# Supplementary material for: The systematic relationships and biogeographic history of ornithischian dinosaurs
Source: PeerJ. 2015 Dec 22;3:e1523. doi: 10.7717/peerj.1523 (PMC4690359; doi:10.7717/peerj.1523)
Supplement: Table S2 — Abbreviations: B, Butler (2005); Bea, Butler, Upchurch & Norman (2008); M, McDonald et al. (2010); N, Nesbitt et al. (2010); S, Scheetz (1999); W, Weishampel et al. (2003); WH, Weishampel & Heinrich (1992); X, Xu et al. (2006). [file peerj-03-1523-s002.docx]

Supplementary Table 2 **Citations for each character and descriptions of how each character was modified from prior studies, if applicable.** Abbreviations: B = Butler (2005); Bea = Butler et al. (2008a); M = McDonald et al. (2010); N =Nesbitt et al. (2010); S = Scheetz (1999); W = Weishampel et al. (2003); WH = Weishampel and Heinrich (1992); X = Xu et al. (2006).

| **#** | **Citation** | **Details of Modifications** |
| --- | --- | --- |
|  |  |  |
| **1** | W(1) |  |
| **2** | B(32) |  |
| **3** | X(3) |  |
| **4** | W(7) |  |
| **5** | WH(1); S(28); W(6) | States of Scheetz (1999) used. |
| **6** | B(13) |  |
| **7** | B(2) |  |
| **8** | B(4) |  |
| **9** | B(5) | Modified state 1. |
| **10** | B(14) |  |
| **11** | B(31) |  |
| **12** | New | New character based on personal observations |
| **13** | B(11) |  |
| **14** | S(29) |  |
| **15** | WH(3) |  |
| **16** | WH(4); S(30); W(8); B(12) | The states of Weishampel and Heinrich (1992) were reversed to match those of the other analyses. |
| **17** | WH(5); S(40); W(9) | Scheetz (1999) uses different terminology, but describes the same character states. |
| **18** | B(10) |  |
| **19** | B(17) |  |
| **20** | NEW | New character based on comments in Irmis et al. (2007). |
| **21** | B(18) |  |
| **22** | Bea(13) |  |
| **23** | B(22) | Added state 2. |
| **24** | B(23) |  |
| **25** | WH(9); B(24) | Butler (2005) set the cutoff at 70%, while Weishampel and Heinrich (1992) set it at 80%. The former convention was followed. |

| **#** | **Citation** | **Details of Modifications** |
| --- | --- | --- |
|  |  |  |
| **26** | S(43) |  |
| **27** | S(45) |  |
| **28** | S(48) |  |
| **29** | S(50) |  |
| **30** | WH(8); S(42); W(5); B(19) | Butler (2005) used different terminology, but describes same character states. |
| **31** | WH(11) |  |
| **32** | B(21) |  |
| **33** | WH(14); W(11) | States of Weishampel and Heinrich (1992) used, while those of Weishampel et al. (2003) were reversed. |
| **34** | S(49) |  |
| **35** | W(10) |  |
| **36** | S(14) |  |
| **37** | S(6); W(16) | The states of Weishampel et al. (2003) were used. States of Scheetz (1999) were transformed as follows: 0 = 0; 1 and 2 = 1. |
| **38** | WH(10); S(44); B(20) | The states of Weishampel and Heinrich (1992) were reversed and state 1 was added. |
| **39** | S(46) |  |
| **40** | S(47) |  |
| **41** | S(16); W(17); B(25) |  |
| **42** | WH(15); B(26) | The states of Butler (2005) were used. |
| **43** | S(15) |  |
| **44** | S(3); W(15) | New states defined for this character. |
| **45** | S(4) |  |
| **46** | New | New character based on personal observations. |
| **47** | S(13) | State 2 was added. |
| **48** | S(1) |  |
| **49** | S(9) | States 1 and 2 were combined into state 1. |
| **50** | S(11) |  |

| **#** | **Citation** | **Details of Modifications** |
| --- | --- | --- |
|  |  |  |
| **51** | S(2) |  |
| **52** | S(7) |  |
| **53** | S(8) |  |
| **54** | S(5) |  |
| **55** | S(10) |  |
| **56** | S(12) |  |
| **57** | W(13); B(30) |  |
| **58** | B(29) |  |
| **59** | S(20) |  |
| **60** | S(18) |  |
| **61** | S(19) |  |
| **62** | S(21) | State 2 was added. |
| **63** | S(24) |  |
| **64** | S(26) |  |
| **65** | S(23) |  |
| **66** | WH(12); S(22); W(12); B(28) | The states from Scheetz (1999) were discarded due to incompatibility. |
| **67** | S(25) |  |
| **68** | B(33) | Modified the definitions of the states. |
| **69** | W(18) |  |
| **70** | WH(17); W(19) |  |
| **71** | B(34) |  |
| **72** | WH(18); S(64); W(20) |  |
| **73** | B(35) |  |
| **74** | S(51) | Redefined states, reduced from 5 states to 4 states, then added a new fifth state. |
| **75** | WH(19); W(21) |  |

| **#** | **Citation** | **Details of Modifications** |
| --- | --- | --- |
|  |  |  |
| **76** | S(61) |  |
| **77** | S(63) | Edited state definitions. |
| **78** | B(36) |  |
| **79** | B(37) |  |
| **80** | B(38) |  |
| **81** | W(23) |  |
| **82** | W(22) | States modified to account for different shapes. |
| **83** | S(62) |  |
| **84** | S(67) |  |
| **85** | S(66) |  |
| **86** | Bea(106) |  |
| **87** | B(27) | State 2 was added. |
| **88** | WH(2) |  |
| **89** | W(2) |  |
| **90** | New | New character added based on personal observations. |
| **91** | B(16) |  |
| **92** | New | New character added based on personal observations. |
| **93** | WH(6) |  |
| **94** | WH(7); W(3); B(15) | Followed the states of Butler (2005) by adding a state 2. |
| **95** | W(4) |  |
| **96** | WH(13) |  |
| **97** | S(65); B(39) | States 0 and 1 of Butler (2005) were combined to match those of Scheetz (1999). |
| **98** | WH(16) |  |
| **99** | S(73) |  |
| **100** | S(17) |  |

| **#** | **Citation** | **Details of Modifications** |
| --- | --- | --- |
|  |  |  |
| **101** | S(68) |  |
| **102** | S(69); W(14) | State 1 was added. |
| **103** | New | New character added based on personal observations. |
| **104** | New | New character added based on personal observations. |
| **105** | S(70) |  |
| **106** | S(72) |  |
| **107** | S(74) |  |
| **108** | S(71) |  |
| **109** | S(75) |  |
| **110** | S(76) |  |
| **111** | WH(20) |  |
| **112** | S(27); W(24); B(1) | The states of Butler (2005) were used and state 2 was modified. |
| **113** | New | New character added based on personal observations. |
| **114** | WH(21); S(56); W(33) | The states of Scheetz (1999) were discarded, state 1 of Weishampel and Heinrich (1992) and Weishampel et al. (2003) was modified. |
| **115** | WH(23); S(38) | Used the states of Scheetz (1999). |
| **116** | B(3) |  |
| **117** | S(31) | States were expanded to be more detailed. |
| **118** | WH(25); S(52) |  |
| **119** | S(33) |  |
| **120** | S(35) |  |
| **121** | S(36) |  |
| **122** | S(37) | State 2 was added. |
| **123** | S(39,55); W(29, 35); B(7) |  |
| **124** | S(54) |  |
| **125** | S(41) | State 2 was added. |

| **#** | **Citation** | **Details of Modifications** |
| --- | --- | --- |
|  |  |  |
| **126** | W(25); B(8) | The states were incongruent, so new states were defined for this character. |
| **127** | W(27) |  |
| **128** | W(28) |  |
| **129** | S(34) |  |
| **130** | WH(24); S(57) | The states from Weishampel and Heinrich (1992) were used. |
| **131** | S(32) |  |
| **132** | WH(22); W(26) |  |
| **133** | W(31) |  |
| **134** | B(6) |  |
| **135** | S(59) |  |
| **136** | S(60) | State 2 was added. |
| **137** | W(30) |  |
| **138** | W(32) |  |
| **139** | W(34) |  |
| **140** | B(9) |  |
| **141** | W(36) |  |
| **142** | S(77) |  |
| **143** | New | New character added based on personal observations. |
| **144** | New | New character added based on personal observations. |
| **145** | B(40) |  |
| **146** | S(78) |  |
| **147** | S(80); W(37); B(42) | States 0 and 1 of Scheetz (1999) were combined into state 0 and states 2 and 3 of Scheetz (1999) were combined into state 1. States 0 and 2 of Butler (2005) were combined into state 0. |
| **148** | S(81); W(38); B(43) | Used the states of Butler (2005) and added state 4 based on Varricchio et al. (2007). |
| **149** | S(82) |  |
| **150** | S(83) |  |

| **#** | **Citation** | **Details of Modifications** |
| --- | --- | --- |
|  |  |  |
| **151** | W(39) |  |
| **152** | S(88) |  |
| **153** | S(85) |  |
| **154** | S(87) |  |
| **155** | B(41) |  |
| **156** | S(79) |  |
| **157** | WH(26); W(40); B(44) |  |
| **158** | S(89) |  |
| **159** | W(43); B(46) | Used the states of Weishampel et al. (2003). |
| **160** | S(90) |  |
| **161** | S(91) |  |
| **162** | W(44) |  |
| **163** | New | New character added based on personal observations. |
| **164** | S(92) | State 2 was added. |
| **165** | WH(28); W(45); B(45) | The states of Butler (2005) were discarded. |
| **166** | B(47) |  |
| **167** | WH(29) |  |
| **168** | W(46) | State 2 from Novas et al., 2004 was added. |
| **169** | S(93) |  |
| **170** | S(94) |  |
| **171** | S(95) |  |
| **172** | W(47) |  |
| **173** | S(97); W(49) |  |
| **174** | W(50) |  |
| **175** | W(48) | States modified. |

| **#** | **Citation** | **Details of Modifications** |
| --- | --- | --- |
|  |  |  |
| **176** | W(51) | State 2 was added. |
| **177** | W(52) |  |
| **178** | W(54) |  |
| **179** | WH(30); W(53) |  |
| **180** | S(96) IN PAMT | States modified and split between two characters. |
| **181** | S(96) IN PAMT | States modified and split between two characters. |
| **182** | S(98) |  |
| **183** | B(52) |  |
| **184** | W(57); B(53) |  |
| **185** | W(55) |  |
| **186** | W(56) |  |
| **187** | B(48) |  |
| **188** | B(51) |  |
| **189** | B(50) |  |
| **190** | S(99) |  |
| **191** | B(54) |  |
| **192** | B(49) |  |
| **193** | S(84) |  |
| **194** | B(58) | State 1 was added. |
| **195** | WH(33); S(105) | Used the states of Weishampel and Heinrich (1992) and added state 2. |
| **196** | WH(32) |  |
| **197** | WH(31); S(104); W(63); B(60) | Used the states of Weishampel et al. (2003) and added state 2 of Butler (2005) as state 3. |
| **198** | B(59) |  |
| **199** | N(219) | State 0 was removed. |
| **200** | S(103) |  |

| **#** | **Citation** | **Details of Modifications** |
| --- | --- | --- |
|  |  |  |
| **201** | WH(34); W(60) | State 2 was added. |
| **202** | B(57) |  |
| **203** | W(58); B(55) |  |
| **204** | WH(35); S(102); W(59) | Used approximately the states of Weishampel and Heinrich (1992), but modified their definitions slightly. |
| **205** | S(100); W(61) |  |
| **206** | B(56) |  |
| **207** | S(101); W(62) |  |
| **208** | W(64) |  |
| **209** | W(65) |  |
| **210** | WH(36) |  |
| **211** | S(106) |  |
| **212** | B(61) |  |
| **213** | S(109) |  |
| **214** | W(67); B(64) |  |
| **215** | S(107) |  |
| **216** | S(108) |  |
| **217** | B(63) |  |
| **218** | B(65) |  |
| **219** | W(68) | Redefined states to take the full range of morphological variation in account. |
| **220** | M(125) |  |
| **221** | W(69); B(62) |  |
| **222** | WH(37); S(110); W(70) | States 1 and 2 of Scheetz (1999) were combined to form state 1. |
| **223** | M(127) |  |
| **224** | W(71) |  |
| **225** | N(244) |  |

| **#** | **Citation** | **Details of Modifications** |
| --- | --- | --- |
|  |  |  |
| **226** | W(66); B(66) |  |
| **227** | S(115) |  |
| **228** | S(113) | State 3 was added. |
| **229** | B(67) | State 3 was added. |
| **230** | N(248) |  |
| **231** | S(114) |  |
| **232** | S(111) |  |
| **233** | S(116) |  |
| **234** | S(117) |  |
| **235** | S(118) |  |
| **236** | S(119) |  |
| **237** | N(279) |  |
| **238** | S(120) |  |
| **239** | New | States modified and split between two characters. |
| **240** | S(121) |  |
| **241** | S(122) |  |
| **242** | S(123) |  |
| **243** | B(69) | States 0 and 1 were combined into state 0. |
| **244** | W(74) |  |
| **245** | W(73); B(68) | Constructed new states by combining the states of both prior analyses. |
| **246** | N(295) |  |
| **247** | S(124) |  |
| **248** | W(72) |  |
| **249** | W(75) |  |
| **250** | WH(27); S(86); W(41); B(70) | For Scheetz (1999) state 2 = 1. |
| **251** | B(71) |  |
| **252** | W(42) |  |
| **253** | B(72) |  |
| **254** | B(73) |  |
| **255** | V(125) |  |
