# Supplementary material for: The systematic relationships and biogeographic history of ornithischian dinosaurs
Source: PeerJ. 2015 Dec 22;3:e1523. doi: 10.7717/peerj.1523 (PMC4690359; doi:10.7717/peerj.1523)
Supplement: Table S3 — Abbreviations: a, polymorphic scoring of 0/1; −, character not applicable to taxon. [file peerj-03-1523-s003.docx]

Supplementary Table 3 **Character codings used to evaluate the systematic relationships of ornithischian dinosaurs in the study.** Abbreviations: a = polymorphic scoring of 0/1; - = character not applicable to taxon.

| Taxon | 1 0 | 2 0 | 3 0 | 4 0 | 5 0 |
| --- | --- | --- | --- | --- | --- |
| *Marasuchus* | ?????????? | ?????????? | ?????????? | ?????????? | ?????????? |
| *Silesaurus* | ?0000000-0 | 0?1?1?000- | ?00--??0?? | ?0?1??00?? | ????101??? |
| *Asilisaurus* | ?0???????? | ???????0?? | ?????????? | ?0???????? | ?????02??? |
| *Sanjuansaurus* | ?????????? | ???????00- | 00?????0?? | ?????????? | ?????????? |
| *Herrerasaurus* | 10000000-0 | 0010?0000- | 000--00020 | ?0101000?? | 0100102??0 |
| *Tawa* | 1000000??0 | 00???0000- | 000--00020 | 00000000?? | 00000????? |
| *Pisanosaurus* | ?????????? | ???????011 | ?????????? | ?????????? | ?????????? |
| *Heterodontosaurus* | 0100011110 | 001?010011 | 0010100010 | 00000003?? | 000000000? |
| *Fruitadens* | ????0?111? | ???0??0111 | ?????????? | ?????????? | ?????????? |
| *Echinodon* | ?1??0????? | ??????0011 | ?????????? | ?????????? | ?????????? |
| *Lycorhinus* | ???????11? | ??????0011 | ?0???????? | ?????????? | ?????????? |
| *Tianyulong* | 010001111? | 001010??1? | ??????0?20 | ?0???000?? | ?????????? |
| *Abrictosaurus* | ?10?011110 | 0?1????011 | 00101????0 | ?0???????? | ?????????? |
| *Eocursor* | ?????????? | ????????1? | ?????????? | ?????????? | ?????????? |
| *Lesothosaurus* | 010??01101 | ?110??0110 | 1010?00010 | ?0?000???? | 000?0000?? |
| *Scutellosaurus* | ?1000??10? | 0??????110 | ??????0??? | 0110??00?? | ?1??00?001 |
| *Scelidosaurus* | ?1???0?0?? | 0?1????111 | 0????????0 | ?1???????? | 0????????? |
| *Emausaurus* | ?1???0?0?0 | ??1????111 | 0????????0 | ?1???????? | 0????????? |
| *Stormbergia* | ?????????? | ?????????? | ?????????? | ?????????? | ?????????? |
| *Agilisaurus* | 01001010-? | 0?1?100?11 | 0020011101 | 1?010000?? | 0100100??1 |
| *Hexinlusaurus* | 01???????? | ?????0??11 | ??10110011 | 00?0?0?0?? | 01??000??? |
| *Yandusaurus* | ?1???????? | ????????11 | ?????????? | ?????????? | ????000??? |
| *Leaellynasaura* | ?????????? | ????????11 | ??????0??1 | 0001?000?? | ????000?0? |
| *Jeholosaurus* | 0100001101 | 011?110111 | 0110010021 | 10010001?? | 1100000001 |
| *Yueosaurus* | ?????????? | ?????????? | ?????????? | ?????????? | ?????????? |
| *Othnielosaurus* | ?????????? | ?????????? | ??????0??? | ???1?????? | ?????????? |
| *Parksosaurus* | ???????10? | ??1??0?111 | 0?????00?? | 101100?0?? | 1?0??00??? |
| *T. neglectus* | 1100101101 | 0111100111 | 0120010021 | 10001000?0 | a100000001 |
| *T. assiniboiensis* | ?????????? | ?????????? | ???0?????? | ?????????? | ?????????? |
| *T. garbanii* | ?????????? | ?????????? | ?????????? | ?????????? | ?????????? |
| *Talenkauen* | ?1001?110? | 0???100?11 | ?0???????? | ?????????? | ?????????? |
| *Notohypsilophodon* | ?????????? | ?????????? | ?????????? | ?????????? | ?????????? |
| *Macrogryphosaurus* | ?????????? | ?????????? | ?????????? | ?????????? | ?????????? |

| Taxon | 1 0 | 2 0 | 3 0 | 4 0 | 5 0 |
| --- | --- | --- | --- | --- | --- |
| *Haya* | 0100001101 | 001110?111 | 01200100?1 | ?0010000?? | 1100000??? |
| *Changchunsaurus* | 0100101101 | 0111?00111 | ?1?0??00?? | ?001?002?? | 0101??0??1 |
| *Oryctodromeus* | ?1001?1??1 | 0111??01?? | ??????0??? | ?0?1???0?? | ????0000?? |
| *Zephyrosaurus* | ?10?0?1101 | 01?11?0111 | 11101?10?1 | 10?1?00300 | ???0000000 |
| *Orodromeus* | 010011110? | 0011?00111 | ?110110021 | 0011?00300 | a10?00000? |
| Kaiparowits Oro. | ?1???????? | ?????????? | ?????????? | 2??1???3?? | ?????????? |
| *Koreanosaurus* | ?????????? | ?????????? | ?????????? | ?????????? | ?????????? |
| *Archaeoceratops* | 0110001100 | 101000?111 | 0010100020 | 10100001?? | 0111000?01 |
| *Liaoceratops* | 0110001100 | 1010?1?111 | 00???0002? | 10100002?? | 0110000000 |
| *Yinlong* | 011?001100 | 1?1001?111 | 0010110020 | 10100001?? | 0101111?00 |
| *Stenopelix* | ?????????? | ?????????? | ?????????? | ?????????? | ?????????? |
| *Micropachycephalosaurus* | ?????????? | ?????????? | ?????????? | ??????0??? | ?????00??? |
| *Wannanosaurus* | ?????????? | ?????????? | ??21??0??? | 10?0???1?0 | ?????????? |
| *Hypsilophodon* | 0100011101 | 0110100111 | 0110100111 | 00110000?? | 1100000001 |
| *Atlascopcosaurus* | ?????????? | ????????11 | ?????????? | ?????????? | ?????????? |
| *Qantassaurus* | ?????????? | ?????????? | ?????????? | ?????????? | ?????????? |
| *Anabisetia* | ?????????? | ???????111 | ?0???????? | ?????????? | ?????????? |
| *Gasparinisaura* | 01???????? | ????????11 | 0?100?0111 | ?0010010?? | 0100000111 |
| *Z. robustus* | 110001--01 | 0?1?0?0111 | 0????00021 | ?0?01?10?? | 0110001111 |
| *Z. shqiperorum* | ?1???????? | ?????????? | ?????00021 | ?00010?0?? | ????0??11? |
| *T. dossi* | 11002?110? | 0?1??11?11 | ?010?????1 | 00001000?? | 0100001?1? |
| *T. tilletti* | 1??0?????? | ????1?0??? | ?0???00111 | 0??1100??? | 1???0??0?? |
| *Rhabdodon* | ?????????? | ???0??0??? | ?????????? | ?????????? | ????0????? |
| *Muttaburrasaurus* | ?????????? | 0?1??0??11 | 0?10?001?1 | 00000110?? | 0?0??01??? |
| *Elrhazosaurus* | ?????????? | ?????????? | ?????????? | ?????????? | ?????????? |
| *Dysalotosaurus* | 010020---0 | 0?10011111 | 0010100110 | 1000011010 | 00010011?0 |
| *Dryosaurus* | 010020---0 | 0?10011111 | 0010000010 | 1000011010 | 00010010?0 |
| *Callovosaurus* | ?????????? | ?????????? | ?????????? | ?????????? | ?????????? |
| *Valdosaurus* | ?????????? | ?????????? | ?????????? | ?????????? | ?????????? |
| *Camptosaurus* | 1101?????? | ???00?1??? | ?????00111 | 1??101??01 | 0???0??0?? |
| *Iguanodon* | 1101?????? | ???0??1??? | ??????0121 | ???100??21 | 0???1??1?? |
| *Ouranosaurus* | 110??????? | ???0??1??? | ??????0120 | ???1?0??21 | 0???1??1?? |

| Taxon | 6 0 | 7 0 | 8 0 | 9 0 | 1 0  0 |
| --- | --- | --- | --- | --- | --- |
| *Marasuchus* | ?????????? | ?????????? | ?????????? | ?????????0 | ?????????? |
| *Silesaurus* | ?100??00?? | ??00111--- | --?40??000 | 00?0??1000 | ???0?0000? |
| *Asilisaurus* | ?????????? | ?????????? | ???4?????? | ?????????? | ?????????? |
| *Sanjuansaurus* | ?????????? | ?????????? | ?????????? | ?????????? | ?0???????? |
| *Herrerasaurus* | ??10000010 | 0000??2--- | --010?0000 | 000010?010 | 0010000??0 |
| *Tawa* | ??00?10000 | 0??????--- | --010?0000 | 0000???100 | ?0100000?? |
| *Pisanosaurus* | ?????????? | ?????????? | ??????0011 | ?10000???? | ?????????? |
| *Heterodontosaurus* | 0?00?01000 | 0?000110?0 | 100?001011 | 0100000000 | 10000000?0 |
| *Fruitadens* | ?????????? | ?????????? | ??0?0??0?? | 0????????? | ?????????? |
| *Echinodon* | ?????????? | ?????????? | 1?000?1011 | 0????????? | ?????????? |
| *Lycorhinus* | ?????????? | ?????????? | ?????????? | ?????????? | ?00??????? |
| *Tianyulong* | ?????????? | ???????1?0 | ???01?1011 | 0110??200? | ??000?0??? |
| *Abrictosaurus* | ?????????? | ????????10 | 1-0?0??011 | 01?0?????0 | 0?????0??? |
| *Eocursor* | ???????0?? | ?????????? | ??0???0001 | 01000????? | ??????0??? |
| *Lesothosaurus* | 0000?00000 | 0000010??? | 0010?00001 | ??00010??0 | 0???000??0 |
| *Scutellosaurus* | ?000?00000 | 0?00?1???? | ???0000001 | 0????11?0? | ?????0???0 |
| *Scelidosaurus* | ??????00?? | ?????????? | ??1????101 | ?????11??? | 0????????? |
| *Emausaurus* | ???????0?? | ?????????? | 0?1????101 | ?????11??? | 0????????? |
| *Stormbergia* | ?????????? | ?????????? | ?????????? | ?????????? | ?????????? |
| *Agilisaurus* | 0??0?00000 | 0000011??? | 001?0?1011 | 0110001000 | 1000001??0 |
| *Hexinlusaurus* | ?????0101? | 0?0001???? | ??12010011 | ?????????0 | ?00?00???? |
| *Yandusaurus* | ??00?????? | ?????????? | ?????????? | ?????????? | ?00??????? |
| *Leaellynasaura* | ??00?????? | ??00?1???? | ?????????? | ?????????? | ?????????? |
| *Jeholosaurus* | 0100101010 | 0000011100 | 0012010011 | 1110100000 | 1000001??0 |
| *Yueosaurus* | ?????????? | ?????????? | ?????????? | ?????????? | ?????????? |
| *Othnielosaurus* | ????????00 | 0??00????? | ?0?1?10?11 | ?????????? | ????????10 |
| *Parksosaurus* | ???01????? | 0????????? | ?????0?011 | 01?0?????0 | 1011001?00 |
| *T. neglectus* | 1110101011 | 0?10111000 | 0112011011 | 111012?000 | 1010001100 |
| *T. assiniboiensis* | ???????010 | 001011???? | ?????????? | ?????????? | ????????10 |
| *T. garbanii* | ?????????? | ?????????? | ?????????? | ?????????? | ?????????? |
| *Talenkauen* | ?????????? | ???????100 | 011?0?1?11 | ?????????? | ?????????? |
| *Notohypsilophodon* | ?????????? | ?????????? | ?????????? | ?????????? | ?????????? |
| *Macrogryphosaurus* | ?????????? | ?????????? | ?????????? | ?????????? | ?????????? |

| Taxon | 6 0 | 7 0 | 8 0 | 9 0 | 1 0 0 |
| --- | --- | --- | --- | --- | --- |
| *Haya* | 1??0?01011 | 0?00110000 | 0111111011 | ?1?00??000 | ?110001??0 |
| *Changchunsaurus* | ???000?000 | 0??????100 | 01111?1011 | 1100120000 | ?0??001??0 |
| *Oryctodromeus* | 01000?10?? | ?????????? | ??1??1??11 | ????1????? | ????00??00 |
| *Zephyrosaurus* | 0100111011 | 00001????? | ????0???11 | ???0??1??? | 0???00?110 |
| *Orodromeus* | 0110111011 | 01000????? | ??12111011 | 0100102?00 | 1000001100 |
| Kaiparowits Oro. | ??????1??? | ?000?????? | ??1?????11 | ?????????? | ?????0???? |
| *Koreanosaurus* | ?????????? | ?????????? | ?????????? | ?????????? | ?????????? |
| *Archaeoceratops* | 0010?0?101 | 0?00101110 | 0110?11011 | 1100002000 | 00?00010?? |
| *Liaoceratops* | 0000?01101 | 0?00001100 | 0110111011 | 0100?0?000 | ????0010?0 |
| *Yinlong* | 0?00?0?10? | 0?00001100 | 0110011011 | 01001??000 | 1011000??0 |
| *Stenopelix* | ?????????? | ?????????? | ?????????? | ?????????? | ?????????? |
| *Micropachycephalosaurus* | ?0???????? | ?????????? | ?????????? | ??????2??? | ?????????? |
| *Wannanosaurus* | ?????0?10? | 0?10?????? | ????0??011 | 01?000???? | ?????01??? |
| *Hypsilophodon* | 0000?00000 | 0000011110 | 0012011011 | 0110100000 | 11000111?0 |
| *Atlascopcosaurus* | ?????????? | ?????????? | ????1???11 | ?????????? | ?????????? |
| *Qantassaurus* | ?????????? | ?????????? | ??12011011 | 0????????? | ?????????? |
| *Anabisetia* | ?????????? | ?????????? | ????1???11 | ?????????? | ?????????0 |
| *Gasparinisaura* | 0????00000 | 0?0?0????? | ??120?1011 | ?100100??0 | 101?001??0 |
| *Z. robustus* | 0010?01001 | 1001102110 | 01101?1011 | 111010201? | ?01?101??0 |
| *Z. shqiperorum* | ?011?0?00? | 10?111??1? | ??10101011 | 11?0102??? | ????10???0 |
| *T. dossi* | 0??1?010?? | 10?01??111 | ?0121?1011 | 11?01??010 | 10111010?0 |
| *T. tilletti* | 00?1001??? | ?1001?1?01 | ????101??? | 1?0?12?11? | ??1?11?0?0 |
| *Rhabdodon* | 000?0????? | ?????????? | ????111?11 | 1??110???? | ????????0? |
| *Muttaburrasaurus* | 1??0???0?? | ??1?10???? | ????????11 | ????1????1 | ?0????1??0 |
| *Elrhazosaurus* | ?????????? | ?????????? | ?????????? | ?????????? | ?????????? |
| *Dysalotosaurus* | 101010100? | ??000?1111 | 0111011011 | 110110?110 | 1011001000 |
| *Dryosaurus* | 101000100? | ?1000?1111 | 0112111011 | 110100?110 | 1011001000 |
| *Callovosaurus* | ?????????? | ?????????? | ?????????? | ?????????? | ?????????? |
| *Valdosaurus* | ?????????? | ?????????? | ?????????? | ?????????? | ?????????? |
| *Camptosaurus* | 1011011?00 | 11101?2?01 | ?1??111??? | 1?111??11? | ??1?00?011 |
| *Iguanodon* | 1011001?0? | 11111?2?01 | ?1??110??? | 1?211???1? | ????0???11 |
| *Ouranosaurus* | 1001?0??00 | 11111?2??? | ?0???10??? | ??111????? | ????????11 |

| Taxon | 1 1 0 | 1 2 0 | 1 3 0 | 1 4 0 | 1 5  0 |
| --- | --- | --- | --- | --- | --- |
| *Marasuchus* | ?????????0 | ?????????? | ?????????? | ?????????? | ???1?0?0?? |
| *Silesaurus* | ?0??000?20 | 01001?0-00 | 01?-3?0-10 | 030003?--- | 0021002100 |
| *Asilisaurus* | ?????????? | ???0???-?? | ???-3????0 | ??0003?--- | ???100?00? |
| *Sanjuansaurus* | ?????????? | ????-?0-?1 | 0?0?2?001? | 04?0?????- | 00??0?000? |
| *Herrerasaurus* | ?0010????? | 0100-10-?1 | 0?0-210010 | 0410?21--- | 00?10000?? |
| *Tawa* | ?0???????? | 0200-10-?1 | 0?0-200010 | 0410?2?--- | ???10??0?? |
| *Pisanosaurus* | ?????????? | ??????1??0 | ??????10?? | 100???1?00 | ?????0???? |
| *Heterodontosaurus* | 0?100?0?20 | 02110120?1 | 1?11001110 | 1?11000?11 | 0?100002?? |
| *Fruitadens* | ?????????? | 0210?01??0 | ??0-0?0001 | 1?0??0?00? | ?0?????2?? |
| *Echinodon* | ?????????? | 020??11-?0 | ??0?0?0001 | 1200?0??0? | ?????????? |
| *Lycorhinus* | ?????????? | ??1?-?1??0 | 0???0?000? | 13?1?????? | ?????????? |
| *Tianyulong* | ?????????? | 0211?11??0 | ????0?0001 | 1????0??0? | ?????????? |
| *Abrictosaurus* | ?????????? | 0200-11??0 | ??1?000001 | 13?1?01?01 | ???????1?? |
| *Eocursor* | 1????????? | ??0??????? | ??0??????1 | ??0000?00? | ???????1?? |
| *Lesothosaurus* | 1?1000?010 | 0000-01100 | 0000010?01 | 0??000?0?0 | ????0????? |
| *Scutellosaurus* | ?????????? | 00000010?0 | 000-0?0001 | 0200002000 | 0011?011?? |
| *Scelidosaurus* | ?????????? | ?????0???? | ??0??????? | ???0?????0 | ????0????? |
| *Emausaurus* | ?????????? | ?????0???? | ??0??????? | ???0?????0 | ?????????? |
| *Stormbergia* | ?????????? | ?????????? | ?????????? | ?????????? | ?0??0????? |
| *Agilisaurus* | 00??1????? | 01000010?0 | 0?0???1001 | 1200?02000 | 01??1?11?? |
| *Hexinlusaurus* | ?????????? | ???0-?1-?0 | 0?000?1001 | 1300?0200? | 00101011?? |
| *Yandusaurus* | ?????????? | ????-?1-?0 | 0?0001100? | 13?0????0? | ?0???????? |
| *Leaellynasaura* | ?????????? | ??????1??? | 11??0?110? | 13?0?????? | ?????????? |
| *Jeholosaurus* | ?0001?1?00 | 0001-010?1 | ????011001 | 1200?01000 | 0010?11200 |
| *Yueosaurus* | ?????????? | ?????????? | ?????????? | ?????????? | 0010?????? |
| *Othnielosaurus* | ????00?1?? | ???1-??000 | 0?000?1001 | ?200001?0? | 0010??120? |
| *Parksosaurus* | ?????1???0 | ???1??10?0 | ???00?10?1 | 1?0?10?00? | ?0?1?0???? |
| *T. neglectus* | ?1011??001 | 0001-01010 | 0100011001 | 1200002110 | 001110?3?0 |
| *T. assiniboiensis* | 0101111000 | ?????????? | ?????????? | 1????????? | ?0???????? |
| *T. garbanii* | ?????????? | ?????????? | ?????????? | ?????????? | ?????????? |
| *Talenkauen* | ?????????? | 0?01??1??? | ????0?11?? | 1???????1? | 00?10?2??? |
| *Notohypsilophodon* | ?????????? | ?????????? | ?????????? | ?????????? | ?01?????00 |
| *Macrogryphosaurus* | ?????????? | ?????????? | ?????????? | ?????????? | 10?10102?0 |

| Taxon | 1 1 0 | 1 2 0 | 1 3 0 | 1  4 0 | 1 5 0 |
| --- | --- | --- | --- | --- | --- |
| *Haya* | 00101????? | 0101-010?? | 020?011001 | 1?00?01?10 | 00?0???2?? |
| *Changchunsaurus* | ????1???1? | 0100-010?0 | 1?0?0?1001 | 1200?0?01? | 0010??1??? |
| *Oryctodromeus* | 00??11?1?? | 0?00???000 | 0??????0?1 | ??00?0?01? | ?021?11310 |
| *Zephyrosaurus* | 0?10111120 | 01???01000 | 010001100? | 12?00????? | ?020?0???? |
| *Orodromeus* | 101011111? | 010??01000 | 00000???01 | 12?000???? | 0020101300 |
| Kaiparowits Oro. | ???0?????? | ??00??1000 | 00??0?0?01 | 1?0000?01? | ?021?????? |
| *Koreanosaurus* | ?????????? | ?????????? | ?????????? | ?????????? | ?021?0??0? |
| *Archaeoceratops* | ?0??1????? | 0201?01??1 | 1?1?0??101 | 141??0??10 | ???0???2?0 |
| *Liaoceratops* | ?0101????1 | 0201?01??1 | ??110?1001 | 1?00?01?10 | ?????????? |
| *Yinlong* | ??101???21 | 020?001??0 | 0???010?0? | 12?0?????? | ?????????? |
| *Stenopelix* | ?????????? | ?????????? | ?????????? | ?????????? | ???????2?? |
| *Micropachycephalosaurus* | ?????????? | ?????????? | ?????????? | ???0?????? | ?????????0 |
| *Wannanosaurus* | ?????????? | ???0???1?? | ?????????0 | ??00?00?0? | ?0?0?????? |
| *Hypsilophodon* | 0010110010 | 0001-02001 | 1111001001 | 1000101010 | 0010101200 |
| *Atlascopcosaurus* | ?????????? | ??????2011 | 11?00?111? | 1?0010?11? | ?????????? |
| *Qantassaurus* | ?????????? | ???????0?? | ???0?????0 | ?400100010 | ?????????? |
| *Anabisetia* | ????0????? | ???1-?1-?1 | ??1?0??11? | 1??0????0? | ?????????? |
| *Gasparinisaura* | 10100??2?0 | ??0???1??1 | 1?1?00111? | 10?0?000?0 | ??1????210 |
| *Z. robustus* | 00200??1?0 | 1-011-2011 | 11111?1000 | 110011011? | ?010?0?320 |
| *Z. shqiperorum* | ?0200????? | ??01-??0?1 | 1111?????0 | 1100110110 | ?0?0?0?32? |
| *T. dossi* | 1???1????0 | 0201???011 | 1111?0001? | 11?0110010 | 11?0?11210 |
| *T. tilletti* | 1???10020? | 1?01???011 | 1111??001? | 11?0110010 | 11?0?11210 |
| *Rhabdodon* | ????100??? | ???????0?1 | 1111??101? | 11?01??11? | ?0???1??10 |
| *Muttaburrasaurus* | ?0??1????? | ???1??10?1 | 1???0?11?? | 1??0????1? | 011??1?2?? |
| *Elrhazosaurus* | ?????????? | ?????????? | ?????????? | ?????????? | ?????????? |
| *Dysalotosaurus* | 0?11110221 | 1-0??-21?? | ??11?11110 | 11?0110010 | 011??112?0 |
| *Dryosaurus* | 0?11100201 | 1-011-2111 | 11111?1110 | 11?0111010 | ?111?1?210 |
| *Callovosaurus* | ?????????? | ?????????? | ?????????? | ?????????? | ?????????? |
| *Valdosaurus* | ?????????? | ?????????? | ?????????? | ?????????? | ?121?????? |
| *Camptosaurus* | 0???001200 | 1?0????111 | 1111??011? | 11??1?100? | ?0???1??21 |
| *Iguanodon* | 1???000220 | ??0????111 | 1111??011? | 11??1?200? | ?1???1??21 |
| *Ouranosaurus* | 1???00002? | ??0????1?? | ???1?????? | 1???1????? | ?????1???? |

| Taxon | 1 6 0 | 1 7 0 | 1 8 0 | 1 9 0 | 2 0  0 |
| --- | --- | --- | --- | --- | --- |
| *Marasuchus* | ???1????0? | ????0????? | ?????????? | ?0001002-? | 00?02---01 |
| *Silesaurus* | 1??????000 | 0???0?020? | 00???????? | ?0000002?0 | 00002---01 |
| *Asilisaurus* | 00?????000 | 0????0???? | ?????????? | ?00000???? | ???02---0? |
| *Sanjuansaurus* | ???????000 | 01??????2? | ?????????? | ??11?????? | ???02---?? |
| *Herrerasaurus* | ?0?????00? | 0????0??2? | 0000?00002 | 20110002?0 | 00002---10 |
| *Tawa* | ???????00? | 0?????01?? | ??00?0?011 | ?0010?02?? | ?0?02---11 |
| *Pisanosaurus* | ?????????? | ?????????? | ?????????? | ??0??????? | 0???????11 |
| *Heterodontosaurus* | 0000100001 | ????000120 | 0000000000 | 001100101? | 00?2000010 |
| *Fruitadens* | ?????????? | ?????????? | ?????????? | ?????????? | ?????????? |
| *Echinodon* | ?????????? | ?????????? | ?????????? | ?????????? | ?????????? |
| *Lycorhinus* | ?????????? | ?????????? | ?????????? | ?????????? | ?????????? |
| *Tianyulong* | ?????????? | ??????102? | ?????????? | ?????????? | ???2?????? |
| *Abrictosaurus* | ?????????? | ????0000?? | ??????0??? | ??1100101? | 00???????? |
| *Eocursor* | ???????00? | ????0?00?? | ??????0??? | ?01100101? | 00?2000010 |
| *Lesothosaurus* | ?0??1????1 | 0????1??00 | 0?00?0???? | 1000001000 | 00?2000010 |
| *Scutellosaurus* | 0??????0?1 | 000??0000? | ?00???00?0 | ??0100100? | 00?200??11 |
| *Scelidosaurus* | ????0?0??? | ?????0???? | ?????????? | ?001??110? | 00?????0?? |
| *Emausaurus* | ?????????? | ?????????? | ?????????? | ?????????? | ?????????? |
| *Stormbergia* | 00??1??001 | 000??1??1? | ?1???????? | ?001001001 | 00?20000?? |
| *Agilisaurus* | 0?1?1?0001 | 000?01100? | 0000??000? | ?101001001 | 01?21?2110 |
| *Hexinlusaurus* | 002????001 | 0???0?1000 | ?000?0000? | ?010001011 | 01?2002110 |
| *Yandusaurus* | ???????010 | 00??0?1??? | ?????????? | ?????????? | ?????????? |
| *Leaellynasaura* | ?????????? | ?????????? | ?????????? | ?????????? | ???2003?10 |
| *Jeholosaurus* | 001????00? | ?0??010000 | 000??????? | ?010001011 | 110200?110 |
| *Yueosaurus* | ?0?????00? | ????0?002? | ?????????? | ?????????? | ?????????? |
| *Othnielosaurus* | ?11???1001 | 00??0?100? | 0?00?00??0 | 0010001011 | 1102002110 |
| *Parksosaurus* | 001???100? | 00000???1? | 00???????? | ???001?011 | 1??2????11 |
| *T. neglectus* | 111??0100? | 0??2001000 | 0000?00000 | 0010101011 | 1112002110 |
| *T. assiniboiensis* | ?1???2???? | ?????????? | ?????????? | ?010?11?11 | 11?21021?? |
| *T. garbanii* | ?????????? | ?????????? | ?????????? | ?????????? | ?????????? |
| *Talenkauen* | ?????????? | ????0??2?? | ?????????? | ?0?01010?? | 1??20?1??? |
| *Notohypsilophodon* | ???????0?? | 0?????120? | ?????????? | ?????????? | ?????????? |
| *Macrogryphosaurus* | ??????1??? | ???????2?? | ?????????? | ?010101?11 | 11120011?? |

| Taxon | 1 6 0 | 1 7 0 | 1 8 0 | 1  9 0 | 2 0 0 |
| --- | --- | --- | --- | --- | --- |
| *Haya* | 0111??0001 | 01000?001? | ???0?????? | ?01000121? | 11220021?? |
| *Changchunsaurus* | ??????000? | 0?00??10?? | ?????????? | ??1??????? | ?????????? |
| *Oryctodromeus* | ?121?2?101 | 001??01010 | 00???????? | ?1100012?1 | 11220021?? |
| *Zephyrosaurus* | ?11??0?1?1 | 00???????0 | ?????????? | ????001??? | ??2??????? |
| *Orodromeus* | 0010?0?101 | 000???1111 | 000???0000 | ?010001211 | 1122002?10 |
| Kaiparowits Oro. | ???????1?? | 0?1???111? | ??00000001 | ?????????? | ??2??????? |
| *Koreanosaurus* | ??????0111 | 01000?1010 | ?????????? | ??1?????11 | 1???????11 |
| *Archaeoceratops* | 011??????? | ?????????? | ?????????? | ?010011011 | 11?20030?? |
| *Liaoceratops* | ?????????? | ?????????? | ?????????? | ?????????? | ?????????? |
| *Yinlong* | ?????????? | ?????????? | ?????????? | ?01011101? | ?1?20030?? |
| *Stenopelix* | ????????0? | ?????????? | ?????????? | ?01000101? | 01?20?3?1? |
| *Micropachycephalosaurus* | ?????????? | ?????????? | ?????????? | ??1??01??? | ?1???????? |
| *Wannanosaurus* | ?????????? | ??????10?? | ?????????? | ??1???1??? | ?????????? |
| *Hypsilophodon* | 0111101001 | 1000001010 | 100000000? | ?010001011 | 1112012110 |
| *Atlascopcosaurus* | ?????????? | ?????????? | ?????????? | ?????????? | ?????????? |
| *Qantassaurus* | ?????????? | ?????????? | ?????????? | ?????????? | ?????????? |
| *Anabisetia* | ???????001 | 00??0?02?? | ?0???????? | ?01010101? | ?1?2001111 |
| *Gasparinisaura* | ?1101??0?1 | 00?????10? | 00???????? | ?010101011 | 1102002111 |
| *Z. robustus* | 101????00? | 0?????110? | 1????????? | ?1110110?? | 1???????11 |
| *Z. shqiperorum* | 1??????000 | 010???110? | 1????????? | ?1110110?1 | 11?2002111 |
| *T. dossi* | 11????0001 | 00001?1100 | 100???10?? | ?11010101? | 11?21?1111 |
| *T. tilletti* | 1110?10001 | 00001?1100 | 100000101? | ?110101011 | 1102011111 |
| *Rhabdodon* | 111????0?0 | 00????1010 | 1????????? | ?1??0????1 | ??2??????1 |
| *Muttaburrasaurus* | ???????00? | 0?????0?10 | 100??????0 | ?1101?1?1? | 11?2??111? |
| *Elrhazosaurus* | ?????????? | ?????????? | ?????????? | ?????????? | ?????????? |
| *Dysalotosaurus* | 1121??00?0 | 10?0???110 | 10????10?? | ?110101011 | 1112111110 |
| *Dryosaurus* | 1120?100?0 | 1?????0110 | 00?0??10?? | ?110101011 | 1102111110 |
| *Callovosaurus* | ?????????? | ?????????? | ?????????? | ?????????? | ?????????? |
| *Valdosaurus* | ?????????? | ?????????? | ?????????? | ????????1? | ???2?12??? |
| *Camptosaurus* | 1111?000?1 | 10????0110 | 1111??101? | ?1?000???1 | ??0??1???1 |
| *Iguanodon* | 1111?200?2 | 10?????121 | 0111??111? | ?1?010???1 | ??1??????1 |
| *Ouranosaurus* | ?????2?0?2 | 1???????21 | 0?1??????? | ?1???????? | ?????????1 |

| Taxon | 2 1 0 | 2 2 0 | 2 3 0 | 2 4 0 | 2 5  0 |
| --- | --- | --- | --- | --- | --- |
| *Marasuchus* | 2?0-0?00?? | 000000001? | 00-000?230 | -0?0020-0? | ??002000?0 |
| *Silesaurus* | 000-01001? | 00-000001? | 00-011?210 | 00?0020-?? | ??0011??00 |
| *Asilisaurus* | ??0-0110?? | ?0?000000? | 0??01??210 | 0??0020-?? | ?????1???0 |
| *Sanjuansaurus* | ?????????0 | 10??????1? | 00-001?20? | 0??0021-?? | ?????????0 |
| *Herrerasaurus* | 0?0-100000 | 100000001? | 00-0011201 | 00?0021-10 | ??00210000 |
| *Tawa* | ????1??000 | 10??00001? | 0???0??211 | 0??0021-?? | ??00?10000 |
| *Pisanosaurus* | ?????0???? | ?????????? | ????????11 | 0???001??? | ????????0? |
| *Heterodontosaurus* | 000-011000 | 000101212? | 00-000??21 | 00??????00 | 000??10000 |
| *Fruitadens* | ???????0?? | ?0?101212? | 00-00???21 | 0???0010?? | ?????????? |
| *Echinodon* | ?????????? | ?????????? | ?????????? | ?????????? | ?????????? |
| *Lycorhinus* | ?????????? | ?????????? | ?????????? | ?????????? | ?????????? |
| *Tianyulong* | 010?0?0??? | ?????????? | ?1000????? | ?????????? | ??0??10001 |
| *Abrictosaurus* | ??0??????? | ???0?1112? | 00-??0??2? | ?????????? | ??0001?00? |
| *Eocursor* | 010-00?000 | 00?0001020 | 00-0001121 | 000??????? | ?????????? |
| *Lesothosaurus* | 010?00000? | 0000001020 | 0????0??2? | ?0?3?01011 | 0?????0??? |
| *Scutellosaurus* | ?10?000000 | 0000001020 | 00-000??21 | 00?0?????? | ??0???00?? |
| *Scelidosaurus* | ?00??1???? | ?0?0?010?? | 0????1??2? | ?????????? | ?????????? |
| *Emausaurus* | ?????????? | ?????????? | ?????????? | ?????????? | ?????????? |
| *Stormbergia* | 0110010000 | 00000010?? | 00-0000321 | 0???001??? | ???0????0? |
| *Agilisaurus* | 011000000? | 100001102? | 00-0001?2? | ?000?????? | ??00010000 |
| *Hexinlusaurus* | 0010??0001 | 000000102? | 00-0001221 | 0100001?11 | 1100110000 |
| *Yandusaurus* | ?????????? | ?????????? | ?0-00????? | ?1???????? | ????????0? |
| *Leaellynasaura* | 00100?0000 | ?0?101202? | 0??000??21 | 0??00010?? | ??0001000? |
| *Jeholosaurus* | 0?1?000010 | 110001212? | 00-0001321 | 0?00001111 | 1100??000? |
| *Yueosaurus* | 0??????00? | 11??012120 | 0???????2? | ?????????? | ??0????00? |
| *Othnielosaurus* | 1?100?0001 | 110001212? | 00-00?0?21 | 01?2101111 | 110001000? |
| *Parksosaurus* | 2011010010 | 1111012120 | 1???001?21 | 0100001??1 | ??00010001 |
| *T. neglectus* | 0011010010 | 111001212? | 1100010221 | 0200001111 | 1000010001 |
| *T. assiniboiensis* | ???????010 | 11100121?0 | 1100010221 | 0?00001?11 | ?000010001 |
| *T. garbanii* | ?????????? | ?????????? | ?100??02?1 | 0?00001?11 | 100001000? |
| *Talenkauen* | ????1??0?? | ?????12?2? | 1????1???? | ?????????? | ??0??1???? |
| *Notohypsilophodon* | ?????????? | 11?00121?? | ?0-00???21 | 0??0?????? | ????????0? |
| *Macrogryphosaurus* | ?0??111??? | ?????????? | ?????????? | ?????????? | ?????????? |

| Taxon | 2 1 0 | 2 2 0 | 2 3 0 | 2  4 0 | 2 5 0 |
| --- | --- | --- | --- | --- | --- |
| *Haya* | 00100??000 | 111001212? | 00-000???? | ????0?1?11 | ???0010000 |
| *Changchunsaurus* | ?????????0 | 11?00121?? | ???????3?1 | 0????????? | ??0??1000? |
| *Oryctodromeus* | ???????001 | 111001212? | 0100000?21 | 0?100????? | ??00010001 |
| *Zephyrosaurus* | 101?1????1 | 1?10012??? | ???00?0?2? | ?110021011 | 100??1000? |
| *Orodromeus* | 00100?0000 | 111?01212? | 01000000?1 | 0112001011 | 1000010000 |
| Kaiparowits Oro. | ???????000 | 111?01212? | ?0-00??2?1 | 001????011 | 110001000? |
| *Koreanosaurus* | ???????001 | 111001212? | 00-00013?1 | 011??????? | ?????????? |
| *Archaeoceratops* | ??0??????1 | 110?0121?? | ????????2? | ???0011011 | 1?0??1000? |
| *Liaoceratops* | ?????????? | ?????????? | ?????????? | ?????????? | ?????????? |
| *Yinlong* | ???????0?? | ????01212? | 0????????? | ?????????? | ??0???000? |
| *Stenopelix* | 110-010??? | ?1?????1?? | ????????2? | ?????????? | ??000?0000 |
| *Micropachycephalosaurus* | ??????0001 | 110?0121?0 | 0??0???3?1 | 0????????? | ?????????? |
| *Wannanosaurus* | ???????00? | ?????1???? | 00???00??? | ?????????? | ?????????? |
| *Hypsilophodon* | 0011010001 | 11000121?0 | 0??0000321 | 0201001111 | 1100110001 |
| *Atlascopcosaurus* | ?????????? | ?????????? | ?????????? | ?????????? | ?????????? |
| *Qantassaurus* | ?????????? | ?????????? | ?????????? | ?????????? | ?????????? |
| *Anabisetia* | 0010??100? | 110111212? | 010000???? | ?????????? | ??000100?1 |
| *Gasparinisaura* | ?01?01?00? | 111111212? | 00-0000221 | 0100021112 | 1101-1110? |
| *Z. robustus* | 100-110110 | 1110012120 | 1101011?21 | 01?00210?? | ???1?????? |
| *Z. shqiperorum* | 100-111110 | 1110012120 | 1111011?21 | 0??0???0?? | ?????????? |
| *T. dossi* | 1010011100 | 110001212? | 1111011221 | 030??????1 | 1?00110001 |
| *T. tilletti* | 0010000100 | 1100012120 | 1111010221 | 0300021111 | 100a010001 |
| *Rhabdodon* | 0?0?1?111? | 110001???0 | 0??1?10??? | 01?012???2 | 01?1-?1??? |
| *Muttaburrasaurus* | ???????1?0 | 1100012120 | 110101??21 | ?1?0101?1? | ??01-1??0? |
| *Elrhazosaurus* | ???????000 | 11000121?1 | 01200????? | ?0???????? | ?????????? |
| *Dysalotosaurus* | 10101?1010 | 1100112121 | 011101??21 | 03?31?11?? | 0?00?11101 |
| *Dryosaurus* | a0101?1010 | 1100112121 | 0101010021 | 03?3101111 | 11?0011101 |
| *Callovosaurus* | ???????000 | 1100011121 | 01110????? | ?????????? | ?????????? |
| *Valdosaurus* | 001?11?000 | 1100012121 | 0121000221 | 130???1011 | ?011--1-?? |
| *Camptosaurus* | a?1?1?1100 | 1?0001???0 | 1111?11??? | 0?0012???? | 0??1??100? |
| *Iguanodon* | 1?1?1?110? | 1?0101???0 | 1131?11??? | 15?012???2 | 01?1??111? |
| *Ouranosaurus* | ????1?1??? | 0?0?01???0 | ??????1??? | 140012???2 | ??????1??? |

| Taxon | 2 5 5 |
| --- | --- |
| *Marasuchus* | 0-0?? |
| *Silesaurus* | 0-000 |
| *Asilisaurus* | 0-0?? |
| *Sanjuansaurus* | 0-??? |
| *Herrerasaurus* | 0-000 |
| *Tawa* | 0-000 |
| *Pisanosaurus* | ??00? |
| *Heterodontosaurus* | 1-00? |
| *Fruitadens* | ????? |
| *Echinodon* | ???0? |
| *Lycorhinus* | ????? |
| *Tianyulong* | 1000? |
| *Abrictosaurus* | ??00? |
| *Eocursor* | ??00? |
| *Lesothosaurus* | 1?00? |
| *Scutellosaurus* | 1?10? |
| *Scelidosaurus* | 1?11? |
| *Emausaurus* | ??10? |
| *Stormbergia* | 1?0?? |
| *Agilisaurus* | 11000 |
| *Hexinlusaurus* | 0-00? |
| *Yandusaurus* | ????? |
| *Leaellynasaura* | ????? |
| *Jeholosaurus* | 1100? |
| *Yueosaurus* | ????? |
| *Othnielosaurus* | ??00? |
| *Parksosaurus* | 1000? |
| *T. neglectus* | 10001 |
| *T. assiniboiensis* | 1?00? |
| *T. garbanii* | ????? |
| *Talenkauen* | 1???? |
| *Notohypsilophodon* | ????? |
| *Macrogryphosaurus* | ????? |

| Taxon | 2 5 5 |
| --- | --- |
| *Haya* | 11000 |
| *Changchunsaurus* | 1?001 |
| *Oryctodromeus* | 11001 |
| *Zephyrosaurus* | ??001 |
| *Orodromeus* | ??000 |
| Kaiparowits Oro. | ??0?? |
| *Koreanosaurus* | ??0?? |
| *Archaeoceratops* | ??000 |
| *Liaoceratops* | ????? |
| *Yinlong* | ????0 |
| *Stenopelix* | 1000? |
| *Micropachycephalosaurus* | ????? |
| *Wannanosaurus* | ????? |
| *Hypsilophodon* | 10000 |
| *Atlascopcosaurus* | ????? |
| *Qantassaurus* | ????? |
| *Anabisetia* | ????? |
| *Gasparinisaura* | 1000? |
| *­Z. robustus* | ??000 |
| *Z. shqiperorum* | 1000? |
| *T. dossi* | 1000? |
| *T. tilletti* | 1000? |
| *Rhabdodon* | ??00? |
| *Muttaburrasaurus* | ??00? |
| *Elrhazosaurus* | ????? |
| *Dysalotosaurus* | 10000 |
| *Dryosaurus* | 10000 |
| *Callovosaurus* | ????? |
| *Valdosaurus* | ????? |
| *Camptosaurus* | ?1??? |
| *Iguanodon* | ?1??? |
| *Ouranosaurus* | ????? |
