# Supplementary material for: The systematic relationships and biogeographic history of ornithischian dinosaurs
Source: PeerJ. 2015 Dec 22;3:e1523. doi: 10.7717/peerj.1523 (PMC4690359; doi:10.7717/peerj.1523)
Supplement: Table S4 — Nodes numbers refer to those labeled above the nodes in Fig. 2. For each character change listed below, the character number is given followed by the CI for that character and the specific state change observed at that branch or internode. [file peerj-03-1523-s004.docx]

Supplementary Table 4 **A list of all unambiguously optimized character state changes for the phylogeny of basal ornithischian relationships.** Nodes numbers refer to those labeled above the nodes in Figure 2. For each character change listed below, the character number is given followed by the CI for that character and the specific state change observed at that branch or internode.

Node 2🡪 Node 54

225 1.00 0 → 1
Node 53 🡪*Silesaurus opolensis*

47 0.50 2 → 1
148 0.43 0 → 1
151 0.33 0 → 1

Node 53 🡪*Asilisaurus kongwe*

207 0.17 0 → 1
219 1.00 1 → 0

Node 2 🡪 Node 3

184 0.25 0 → 1
199 1.00 0 → 1
230 1.00 0 → 1
 237 1.00 0 → 1

Node 3 🡪Node 52

120 0.20 0 → 1
133 0.33 0 → 1
205 0.20 0 → 1
211 0.25 0 → 1

Node 52 🡪 Node 53

183 0.33 0 → 1
229 1.00 1 → 0

Node 52 🡪*Tawa hallae*

56 0.33 0 → 1
 88 0.33 0 → 1
 112 0.29 1 → 2
 179 0.50 0 → 1
Node 3 🡪 Node 4

19 1.00 0 → 1
 80 1.00 0 → 1

82 1.00 0 → 1
 117 0.40 0 → 1
 131 0.50 0 → 1
 236 0.33 2 → 0
Node 4 🡪*Pisanosaurus mertii*

127 0.14 0 → 1
Node 4 🡪 Node 5
 200 0.14 1 → 0
 229 1.00 1 → 2
Node 5 🡪 Node 48

183 0.33 0 → 1
Node 48 🡪 Node 49

77 0.25 0 → 1
Node 49 🡪 Node 50

134 1.00 0 → 1
 Node 50 🡪 Node 51

113 1.00 0 → 1
 148 0.43 1 → 2
 214 0.20 0 → 1
 217 0.50 1 → 2
Node 51 🡪*Heterodontosaurus tucki*

15 0.33 1 → 0
 16 0.20 0 → 1
 38 0.50 0 → 3
 68 0.33 1 → 0
 117 0.40 1 → 2
 120 0.20 0 → 1
 121 0.20 0 → 1
 127 0.14 0 → 1
 128 0.17 0 → 1
 129 0.25 0 → 1
 130 0.25 1 → 0
 133 0.33 0 → 1
 139 0.17 0 → 1
 168 0.25 0 → 1
 202 0.20 1 → 0
 207 0.17 0 → 1
 Node 51 🡪*Fruitadens haagarorum*

18 0.50 0 → 1
 116 0.50 1 → 0
Node 51 🡪*Tianyulong confuciusi*

75 0.14 0 → 1
 83 0.25 0 → 1
 167 0.17 0 → 1
 222 0.17 0 → 1
 250 0.25 0 → 1
 Node 5 🡪 Node 6

12 0.25 0 → 1
 18 0.50 0 → 1
 71 1.00 0 → 1

73 1.00 0 → 1
 109 0.25 2 → 1
 116 0.50 1 → 0
 137 0.22 1 → 2
 147 0.50 0 → 1
 240 0.50 0 → 1
Node 6 🡪 Node 45

52 0.50 1 → 0
 86 0.67 0 → 1
 112 0.29 1 → 0
 122 0.67 1 → 0
 131 0.50 1 → 0
Node 45 🡪*Lesothosaurus diagnosticus*

21 0.50 0 → 1
 118 0.33 0 → 1
 184 0.25 1 → 0
 234 0.60 0 → 3
Node 45 🡪 Node 46

32 1.00 0 → 1
 253 1.00 0 → 1
Node 46 🡪 Node 47

8 0.33 1 → 0
 78 1.00 0 → 1
Node 46 🡪*Scelidosaurus harrisonii*

254 1.00 0 → 1
Node 6 🡪 Node 7

190 1.00 0 → 1
 203 0.33 0 → 1
Node 7 🡪*Stormbergia dangershoeki*

169 0.22 0 → 1
 172 0.50 0 → 1
 206 0.17 0 → 1
 227 0.14 1 → 0
Node 7 🡪 Node 8

145 0.50 0 → 1
 192 1.00 0 → 1
 197 0.43 0 → 2
 198 0.50 0 → 1
Node 8 🡪*Agilisaurus louderbacki*

5 0.40 0 → 1
 8 0.33 1 → 0
 23 0.50 1 → 2
 25 0.20 1 → 0
 27 0.50 0 → 1
 28 0.25 0 → 1
 29 0.29 1 → 0
 45 0.20 0 → 1
 77 0.25 0 → 1
 142 0.33 0 → 1
 182 0.33 0 → 1
 195 0.40 0 → 1
Node 8 🡪 Node 9

57 0.25 0 → 1
 183 0.33 0 → 1
 184 0.25 1 → 0
 189 0.50 0 → 1
 202 0.20 1 → 0
 232 0.56 0 → 1
Node 9🡪*Hexinlusaurus multidens*

34 0.13 1 → 0
 210 0.20 0 → 1
 216 0.33 1 → 0
 245 0.40 0 → 1
Node 9 🡪*Yandusaurus hongheensis*

159 0.50 0 → 1
 160 0.40 1 → 0
Node 9 🡪*Leaellynasaura amicagraphica*

121 0.20 0 → 1
 128 0.17 0 → 1
 197 0.43 2 → 3
 214 0.20 0 → 1
Node 9 🡪 Node 10

114 0.20 0 → 1
 137 0.22 2 → 1
 148 0.43 1 → 2
 191 0.50 0 → 1
 212 1.00 0 → 1
 218 0.50 0 → 1
 238 0.20 0 → 1
Node 10 🡪 Node 44

167 0.17 1 → 0
Node 44 🡪*Jeholosaurus shangyuanensis*

209 0.20 0 → 1
Node 44 🡪*Yueosaurus tiantaiensis*

169 0.22 0 → 2
Node 10 🡪 Node 11

152 0.25 0 → 1
 210 0.20 0 → 1
 227 0.14 1 → 0
Node 11 🡪*Othnielosaurus consors*

74 0.38 2 → 1
 99 0.25 0 → 1
 105 0.20 1 → 0
 108 0.40 0 → 1
 157 0.33 0 → 1
 201 0.40 0 → 1
 234 0.60 0 → 2
 235 0.50 0 → 1
Node 11 🡪 Node 12

77 0.25 0 → 1
 139 0.17 0 → 1
Node 12 🡪 Node 33

5 0.40 0 → 1
 14 1.00 0 → 1
 98 0.50 0 → 1
 213 0.33 0 → 1
 242 0.20 1 → 0
 255 0.33 0 → 1
Node 33 🡪 Node 34

23 0.50 1 → 2
 25 0.20 1 → 0
 51 0.50 0 → 1
 86 0.67 0 → 2
 210 0.20 1 → 0
Node 34 🡪 Node 36

55 0.25 0 → 1
 144 0.17 0 → 1
 157 0.33 0 → 1
 204 0.50 0 → 1
 209 0.20 0 → 1

221 0.33 0 → 1
 250 0.25 0 → 1
 252 0.25 1 → 0
Node 36 🡪 New Parksosaurus

76 0.33 1 → 0
 94 0.33 0 → 1
 135 0.50 0 → 1
 139 0.17 1 → 0
 152 0.25 1 → 0
 186 0.25 0 → 1
 200 0.14 0 → 1
 201 0.40 0 → 2
 214 0.20 0 → 1
 227 0.14 0 → 1
Node 36 🡪 Node 37

169 0.22 1 → 0
 185 0.17 0 → 1
 226 0.17 0 → 1
Node 37 🡪 Node 39

222 0.17 0 → 1
Node 39 🡪*Thescelosaurus neglectus*

110 0.33 0 → 1
Node 39 🡪*Thescelosaurus assiniboiensis*

99 0.25 0 → 1
 156 0.40 0 → 2
 186 0.25 0 → 1
 195 0.40 0 → 1
Node 37 🡪 Node 38

145 0.50 1 → 0
 168 0.25 0 → 2
 197 0.43 2 → 1
 205 0.25 0 → 1

Node 79 🡪*Macrogryphosaurus gondwanicus*

141 0.50 0 → 1
Node 34 🡪 Node 35

74 0.38 2 → 1
 75 0.14 0 → 1
Node 35 🡪*Haya griva*

5 0.40 1 → 0
 12 0.25 1 → 0
 68 0.33 1 → 0
 85 0.20 1 → 0
 92 0.50 0 → 1
 167 0.17 1 → 0
 255 0.33 1 → 0
Node 35 🡪*Changchunsaurus parvus*

38 0.50 0 → 2
 44 0.25 0 → 1
 59 0.20 1 → 0
 114 0.20 1 → 0
 121 0.20 0 → 1
Node 33 🡪 Node 40

108 0.40 0 → 1
 114 0.20 1 → 0
 143 0.33 1 → 2
 148 0.43 2 → 3
 158 1.00 0 → 1
 233 1.00 0 → 1
Node 40 🡪 Node 43

144 0.17 0 → 1
Node 43 🡪*Oryctodromeus cubicularis*

146 0.25 0 → 1
 149 0.50 0 → 1
 163 0.50 0 → 1
 222 0.17 0 → 1
Node 43 🡪*Koreanosaurus boseongensis*

159 0.50 0 → 1
 162 0.25 0 → 1
 227 0.14 0 → 1
Node 40 🡪 Node 41

38 0.50 0 → 3
 55 0.25 0 → 1
Node 41 🡪*Zephyrosaurus schaffi*

5 0.40 1 → 0
 27 0.50 0 → 1
 91 0.25 1 → 0
 99 0.25 0 → 1
 109 0.25 1 → 2
 201 0.40 0 → 1
 205 0.25 0 → 1
 236 0.33 0 → 2
Node 41 🡪 Node 42

122 0.67 1 → 0
 210 0.20 1 → 0
Node 42 🡪*Orodromeus makelai*

62 0.33 0 → 1
 222 0.17 0 → 1
Node 42 🡪’Kaiparowits Orodromine’

144 0.17 0 → 1
 163 0.50 0 → 1
 180 0.67 0 → 1
 232 0.56 1 → 0
 242 0.20 0 → 1
Node 12 🡪 Node 13

26 0.33 1 → 0
 52 0.50 1 → 0
 112 0.29 1 → 2
 120 0.20 0 → 1
 123 0.33 0 → 1
 124 0.33 0 → 1
 252 0.25 1 → 0
Node 13 🡪 Node 29

87 0.25 0 → 2
 197 0.43 2 → 3
 203 0.33 1 → 0
Node 29 🡪 Node 30

186 0.25 0 → 1
 Node 30 🡪 Node 32

85 0.20 1 → 0

Node 32 🡪 *Archaeoceratops oshimoi*
 81 0.20 0 → 1
 133 0.33 0 → 1
Node 30 🡪 Node 31

16 0.20 0 → 1

50 0.25 1 → 0

Node 31 🡪*Liaoceratops yanzigouensis*

38 0.50 1 → 2
 75 0.14 0 → 1
Node 31 🡪*Yinlong downsi*

26 0.33 0 → 1
 45 0.20 0 → 1
 46 1.00 0 → 1
 47 0.50 0 → 1
 97 0.50 1 → 0
 120 0.20 1 → 0
 127 0.14 1 → 0

Node 32 🡪*Wannanosaurus yansiensis*

23 0.50 1 → 2
 24 1.00 0 → 1
 63 0.25 0 → 1
 114 0.20 1 → 0
 130 0.25 1 → 0
 139 0.17 1 → 0

Node 13 🡪 Node 14

28 0.25 0 → 1
 29 0.29 2 → 1
 31 0.33 1 → 0
 117 0.40 1 → 2
 126 0.25 1 → 0

132 0.44 2 → 0
 250 0.25 0 → 1
Node 14 🡪*Hypsilophodon foxii*
 83 0.25 0 → 1
 92 0.50 0 → 1
 96 0.50 0 → 1
 98 0.50 0 → 1
 112 0.29 2 → 0
 157 0.33 0 → 1
 161 0.50 0 → 1
 196 0.50 0 → 1

204 0.50 0 → 1
 232 0.56 1 → 2
 234 0.60 0 → 1
 245 0.40 0 → 1
Node 14 🡪 Node 15

49 1.00 0 → 1
 75 0.14 0 → 1
 108 0.40 0 → 2
 119 0.50 0 → 1
 130 0.25 1 → 0
 149 0.50 0 → 1
 200 0.14 0 → 1
 207 0.17 0 → 1
 228 0.38 3 → 2
Node 15 🡪 Node 26

124 0.33 1 → 0
Node 26 🡪*Atlascoposaurus loadsi*

138 0.33 0 → 1
Node 26 🡪 Node 27

117 0.40 2 → 1
Node 27 🡪 Node 28

75 0.14 1 → 0
Node 28 🡪*Qantassaurus intrepidus*

132 0.44 0 → 4
Node 27 🡪*Anabisetia saldivai*

139 0.17 1 → 0
 168 0.43 1 → 2
Node 15 🡪 Node 16

34 0.13 1 → 0
 47 0.50 0 → 1
 61 1.00 0 → 1
 65 0.17 0 → 1
 81 0.20 0 → 1
 132 0.44 0 → 1
 136 1.00 0 → 1
 182 0.33 0 → 1
 208 0.50 0 → 1
 221 0.25 0 → 1
 224 0.50 0 → 1
 226 0.17 0 → 1
Node 16 🡪 Node 24

28 0.25 1 → 0
 29 0.29 1 → 2
 64 0.50 0 → 1
 74 0.38 2 → 0
 105 0.20 1 → 0
 148 0.43 2 → 3
 149 0.50 1 → 2
 184 0.25 0 → 1
 186 0.25 0 → 1
 213 0.33 0 → 1
 227 0.14 0 → 1
 238 0.20 1 → 0
Node 24 🡪*Zalmoxes robustus*

207 0.17 1 → 0

Node 16 🡪 Node 25

37 0.50 1 → 0
 101 0.17 0 → 1
 127 0.14 1 → 0
 142 0.25 0 → 1
 205 0.20 1 → 0
 232 0.56 1 → 3
Node 25 🡪*Tenontosaurus dossi*

17 0.50 0 → 1
 111 0.50 1 → 0
 195 0.40 0 → 1
 227 0.14 0 → 1
 245 0.40 0 → 1
Node 25 🡪*Tenontosaurus tilletti*

34 0.13 0 → 1
 41 0.43 0 → 1
 62 0.33 0 → 1
 69 0.20 1 → 0
 88 0.33 0 → 1
 96 0.50 0 → 1
 206 0.17 1 → 0
 207 0.17 1 → 0
Node 16 🡪*Rhabdodon priscus*

84 0.50 0 → 1
 168 0.25 1 → 0
 193 0.33 0 → 2
 203 0.25 1 → 0
 221 0.25 1 → 0
 235 0.33 0 → 1
Node 16 🡪 Node 17

36 0.50 0 → 1
 51 0.50 0 → 1
 142 0.25 0 → 1
 235 0.33 0 → 1
Node 17 🡪*Muttaburrasaurus longdoni*

90 1.00 0 → 1
 117 0.40 2 → 1
Node 17 🡪 Node 18

31 0.33 0 → 1
 118 0.33 0 → 1
 161 0.50 0 → 1
Node 18 🡪 Node 19

208 0.50 1 → 0
 220 1.00 0 → 1
 221 0.33 1 → 0
Node 19 🡪 Node 21

223 0.50 1 → 2
Node 21 🡪*Elrhazosaurus nigeriensis*

224 0.50 1 → 0
 232 0.56 3 → 0
Node 19 🡪 Node 20

209 0.20 0 → 1
 215 0.50 0 → 1
 244 0.50 1 → 0
Node 20 🡪*Dysalotosaurus lettowvorbecki*

55 0.25 0 → 1
 74 0.38 2 → 1
 75 0.14 1 → 0
 106 0.33 0 → 1
 109 0.25 0 → 2
 193 0.33 0 → 1
Node 20 🡪*Dryosaurus altus*

28 0.25 1 → 0
 85 0.20 1 → 0
 171 0.25 1 → 0
 223 0.50 1 → 0

241 0.33 0 → 1
Node 19 🡪*Callovosaurus leedsi*

217 0.40 2 → 1
Node 18 🡪 Node 22
 4 1.00 0 → 1
 34 0.13 0 → 1
 40 1.00 0 → 1
 54 0.33 0 → 1
 69 0.20 1 → 0
 99 0.25 0 → 1
 100 1.00 0 → 1
 105 0.20 1 → 0
 127 0.14 1 → 0
 139 0.17 1 → 0
 150 1.00 0 → 1
 172 0.50 0 → 1
 174 1.00 0 → 1
 252 0.25 0 → 1
Node 22 🡪*Camptosaurus dispar*

56 0.33 0 → 1
 107 0.33 0 → 1
 142 0.33 1 → 0
 185 0.17 1 → 0
Node 22 🡪 Node 23

29 0.29 1 → 2
 36 0.50 1 → 0
 39 1.00 0 → 2
 45 0.20 0 → 1
 64 0.50 0 → 1
 77 0.25 1 → 0
 101 0.17 0 → 1
 109 0.25 0 → 2
 169 0.22 1 → 2
 170 0.50 0 → 1
 171 0.25 1 → 0
 231 0.50 0 → 1
Node 23 🡪*Iguanodon bernissartensis*

83 0.25 1 → 2
Node 23 🡪*Ouranosaurus nigeriensis*

30 0.25 1 → 0
 53 0.17 1 → 0
 72 0.25 1 → 0
 108 0.40 2 → 0
 211 0.25 1→ 0
