# Supplementary material for: The systematic relationships and biogeographic history of ornithischian dinosaurs
Source: PeerJ. 2015 Dec 22;3:e1523. doi: 10.7717/peerj.1523 (PMC4690359; doi:10.7717/peerj.1523)
Supplement: Table S5 — Node numbers correspond to those labeled in Figs. 3 and 4. Under the ‘Analysis’ column, the label ‘PB’ refers to parsimony-based analysis, ‘LEB’ refers to the likelihood-based analysis where all branch lengths were set equal to each other, and ‘LFR’ refers to the likelihood-based analysis with branch lengths set equal to implied missing fossil records. In each row, the most-likely ancestral area(s) are highlighted in bold text. [file peerj-03-1523-s005.docx]

Supplementary Table 5 **Results from the biogeographical ancestral state reconstructions.** Node numbers correspond to those labeled in Figures 3 and 4. Under the ‘Analysis’ column, the label ‘PB’ refers to parsimony-based analysis, ‘LEB’ refers to the likelihood-based analysis where all branch lengths were set equal to each other, and ‘LFR’ refers to the likelihood-based analysis with branch lengths set equal to implied missing fossil records. In each row, the most-likely ancestral area(s) are highlighted in bold text.

| **Node** | **Analysis** | **Africa** | **South America** | **Asia** | **North America** | **Europe** | **Australia** |
| --- | --- | --- | --- | --- | --- | --- | --- |
| 1 | PB | 0.0 | **100.0** | 0.0 | 0.0 | 0.0 | 0.0 |
|  | LEB | 7.5 | **79.8** | 2.3 | 3.1 | 5.0 | 2.3 |
|  | LFR | **52.5** | 34.8 | 2.8 | 2.8 | 4.2 | 2.8 |
| 2 | PB | 0.0 | **100.0** | 0.0 | 0.0 | 0.0 | 0.0 |
|  | LEB | 15.2 | **68.0** | 2.0 | 4.0 | 8.8 | 2.0 |
|  | LFR | **61.0** | 29.0 | 2.1 | 2.1 | 3.7 | 2.1 |
| 3 | PB | 0.0 | **100.0** | 0.0 | 0.0 | 0.0 | 0.0 |
|  | LEB | 12.0 | **75.5** | 1.4 | 6.3 | 3.4 | 1.4 |
|  | LFR | 0.5 | **99.0** | 0.1 | 0.2 | 0.1 | 0.1 |
| 4 | PB | 0.0 | **100.0** | 0.0 | 0.0 | 0.0 | 0.0 |
|  | LEB | 17.9 | **74.5** | 1.4 | 2.8 | 2.1 | 1.2 |
|  | LFR | 0.5 | **99.2** | <0.1 | 0.1 | <0.1 | <0.1 |
| 5 | PB | **100.0** | 0.0 | 0.0 | 0.0 | 0.0 | 0.0 |
|  | LEB | **80.0** | 12.0 | 2.2 | 1.9 | 2.6 | 1.2 |
|  | LFR | **92.8** | 3.4 | 1.0 | 1.0 | 0.9 | 0.9 |
| 6 | PB | **100.0** | 0.0 | 0.0 | 0.0 | 0.0 | 0.0 |
|  | LEB | **86.1** | 3.9 | 4.1 | 2.3 | 2.5 | 1.0 |
|  | LFR | **97.7** | <0.1 | 1.1 | 0.8 | 0.3 | <0.1 |
| 7 | PB | **100.0** | 0.0 | 0.0 | 0.0 | 0.0 | 0.0 |
|  | LEB | **82.2** | 1.9 | 11.9 | 1.5 | 1.5 | 1.1 |
|  | LFR | **97.2** | <0.1 | 2.2 | 0.4 | 0.1 | <0.1 |
| 8 | PB | 0.0 | 0.0 | **100.0** | 0.0 | 0.0 | 0.0 |
|  | LEB | 6.4 | 0.7 | **90.8** | 0.7 | 0.6 | 0.7 |
|  | LFR | 41.2 | 0.7 | **55.8** | 0.8 | 0.7 | 0.7 |
| 9 | PB | 0.0 | 0.0 | **100.0** | 0.0 | 0.0 | 0.0 |
|  | LEB | 0.4 | <0.1 | **98.2** | 0.4 | <0.1 | 0.7 |
|  | LFR | <0.1 | <0.1 | **99.9** | <0.1 | <0.1 | <0.1 |
| 10 | PB | 0.0 | 0.0 | **100.0** | 0.0 | 0.0 | 0.0 |
|  | LEB | 0.6 | 0.5 | **93.2** | 0.4 | 0.6 | 0.6 |
|  | LFR | 1.0 | 1.0 | **85.7** | 6.3 | 5.0 | 1.1 |

| **Node** | **Analysis** | **Africa** | **South America** | **Asia** | **North America** | **Europe** | **Australia** |
| --- | --- | --- | --- | --- | --- | --- | --- |
| 11 | PB | 0.0 | 0.0 | 50.0 | **50.0** | 0.0 | 0.0 |
|  | LEB | 1.2 | 1.2 | 42.9 | **50.7** | 2.8 | 1.2 |
|  | LFR | 1.0 | 1.0 | **81.1** | 8.8 | 6.9 | 1.2 |
| 12 | PB | 0.0 | 0.0 | 50.0 | **50.0** | 0.0 | 0.0 |
|  | LEB | 1.3 | 1.4 | 41.0 | **48.1** | 6.7 | 1.4 |
|  | LFR | 1.1 | 1.1 | **79.0** | 8.1 | 9.4 | 1.3 |
| 13 | PB | 0.0 | 0.0 | **33.3** | **33.3** | **33.3** | 0.0 |
|  | LEB | 1.9 | 2.1 | **47.4** | 15.8 | 30.1 | 2.5 |
|  | LFR | 1.1 | 1.0 | **77.2** | 7.2 | 12.2 | 1.3 |
| 14 | PB | 0.0 | 0.0 | 0.0 | 0.0 | **100.0** | 0.0 |
|  | LEB | 1.2 | 2.1 | 8.2 | 3.8 | **81.2** | 3.4 |
|  | LFR | 2.1 | 1.9 | **60.6** | 9.1 | 23.7 | 2.6 |
| 15 | PB | 0.0 | 0.0 | 0.0 | 0.0 | **100.0** | 0.0 |
|  | LEB | 1.3 | 4.2 | 3.5 | 2.9 | **79.5** | 8.5 |
|  | LFR | 2.7 | 2.3 | **47.0** | 10.9 | 33.4 | 3.6 |
| 16 | PB | 0.0 | 0.0 | 0.0 | 0.0 | **100.0** | 0.0 |
|  | LEB | 0.2 | 0.2 | 0.2 | 2.2 | **95.9** | 1.4 |
|  | LFR | 3.0 | 2.4 | 34.6 | 12.6 | **44.0** | 3.4 |
| 17 | PB | 0.0 | 0.0 | 0.0 | 0.0 | **100.0** | 0.0 |
|  | LEB | 3.0 | 1.2 | 1.2 | 4.1 | **74.2** | 16.2 |
|  | LFR | 2.9 | 2.1 | 23.2 | 13.6 | **55.3** | 2.8 |
| 18 | PB | 0.0 | 0.0 | 0.0 | 0.0 | **100.0** | 0.0 |
|  | LEB | 7.6 | 1.1 | 1.1 | 8.4 | **76.9** | 5.0 |
|  | LFR | 2.4 | 1.4 | 12.5 | 14.5 | **67.5** | 1.8 |
| 19 | PB | 0.0 | 0.0 | 0.0 | 0.0 | **100.0** | 0.0 |
|  | LEB | 10.7 | 0.4 | 0.4 | 4.4 | **83.3** | 0.9 |
|  | LFR | 1.8 | 0.6 | 4.1 | 8.8 | **84.0** | 0.7 |
| 20 | PB | **33.3** | 0.0 | **33.3** | **33.3** | 0.0 | 0.0 |
|  | LEB | **36.3** | 2.2 | 2.2 | 31.0 | 25.9 | 2.3 |
|  | LFR | 44.7 | 0.7 | 0.9 | **49.4** | 3.5 | 0.7 |

| **Node** | **Analysis** | **Africa** | **South America** | **Asia** | **North America** | **Europe** | **Australia** |
| --- | --- | --- | --- | --- | --- | --- | --- |
| 21 | PB | 0.0 | 0.0 | 0.0 | 0.0 | **100.0** | 0.0 |
|  | LEB | 17.3 | 0.8 | 0.8 | 1.9 | **78.4** | 0.9 |
|  | LFR | 1.8 | 0.9 | 0.9 | 1.0 | **94.6** | 0.9 |
| 22 | PB | 0.0 | 0.0 | 0.0 | 0.0 | **100.0** | 0.0 |
|  | LEB | 11.0 | 1.7 | 1.7 | 24.4 | **58.3** | 2.9 |
|  | LFR | 1.4 | 1.2 | 1.9 | **81.9** | 12.3 | 1.3 |
| 23 | PB | 0.0 | 0.0 | 0.0 | 0.0 | **100.0** | 0.0 |
|  | LEB | 23.6 | 1.6 | 1.6 | 8.1 | **63.3** | 1.9 |
|  | LFR | 3.9 | 1.4 | 1.4 | 3.4 | **88.6** | 1.4 |
| 24 | PB | 0.0 | 0.0 | 0.0 | 0.0 | **100.0** | 0.0 |
|  | LEB | <0.1 | <0.1 | <0.1 | <0.1 | **99.4** | <0.1 |
|  | LFR | <0.1 | <0.1 | <0.1 | <0.1 | **99.9** | <0.1 |
| 25 | PB | 0.0 | 0.0 | 0.0 | **100.0** | 0.0 | 0.0 |
|  | LEB | 0.5 | 0.5 | 0.5 | **91.1** | 6.7 | 0.6 |
|  | LFR | <0.1 | <0.1 | <0.1 | **99.9** | <0.1 | <0.1 |
| 26 | PB | 0.0 | **33.3** | 0.0 | 0.0 | **33.3** | **33.3** |
|  | LEB | 2.3 | 22.4 | 2.9 | 2.7 | 22.9 | **46.7** |
|  | LFR | 3.1 | 3.3 | 3.9 | 3.2 | 3.7 | **82.8** |
| 27 | PB | 0.0 | **50.0** | 0.0 | 0.0 | 0.0 | **50.0** |
|  | LEB | 1.2 | **67.6** | 1.3 | 1.2 | 4.3 | 24.5 |
|  | LFR | 2.2 | 2.5 | 2.7 | 2.2 | 2.5 | **87.9** |
| 28 | PB | 0.0 | **50.0** | 0.0 | 0.0 | 0.0 | **50.0** |
|  | LEB | 1.0 | **65.6** | 1.0 | 1.0 | 1.9 | 29.4 |
|  | LFR | 1.1 | 1.3 | 1.4 | 1.2 | 1.3 | **93.6** |
| 29 | PB | 0.0 | 0.0 | **50.0** | 0.0 | **50.0** | 50.0 |
|  | LEB | 0.7 | 0.8 | **78.0** | 2.5 | 17.2 | 0.8 |
|  | LFR | 1.0 | 1.0 | **85.8** | 3.1 | 8.1 | 1.1 |
| 30 | PB | 0.0 | 0.0 | **100.0** | 0.0 | 0.0 | 0.0 |
|  | LEB | 0.2 | 0.2 | **97.5** | 0.4 | 1.5 | 0.2 |
|  | LFR | 0.7 | 0.7 | **91.0** | 1.9 | 4.8 | 0.8 |

| **Node** | **Analysis** | **Africa** | **South America** | **Asia** | **North America** | **Europe** | **Australia** |
| --- | --- | --- | --- | --- | --- | --- | --- |
| 31 | PB | 0.0 | 0.0 | **100.0** | 0.0 | 0.0 | 0.0 |
|  | LEB | <0.1 | <0.1 | **99.6** | <0.1 | <0.1 | <0.1 |
|  | LFR | 0.4 | 0.4 | **95.8** | 0.9 | 2.1 | 0.4 |
| 32 | PB | 0.0 | 0.0 | **100.0** | 0.0 | 0.0 | 0.0 |
|  | LEB | <0.1 | <0.1 | **99.6** | 0.4 | <0.1 | <0.1 |
|  | LFR | 1.2 | 1.2 | **94.0** | 1.2 | 1.2 | 1.2 |
| 33 | PB | 0.0 | 0.0 | **50.0** | **50.0** | 0.0 | 0.0 |
|  | LEB | 0.8 | 1.0 | 20.8 | **64.6** | 1.9 | 0.9 |
|  | LFR | 1.9 | 2.4 | 42.3 | **49.5** | 1.9 | 1.9 |
| 34 | PB | 0.0 | 0.0 | **50.0** | **50.0** | 0.0 | 0.0 |
|  | LEB | 1.0 | 1.5 | 34.9 | **60.2** | 1.3 | 1.0 |
|  | LFR | 1.8 | 2.6 | **60.7** | 31.3 | 1.8 | 1.8 |
| 35 | PB | 0.0 | 0.0 | **100.0** | 0.0 | 0.0 | 0.0 |
|  | LEB | 0.4 | 0.5 | **94.1** | 4.2 | 0.4 | 0.4 |
|  | LFR | 1.1 | 1.5 | **80.9** | 14.3 | 1.1 | 1.1 |
| 36 | PB | 0.0 | 0.0 | 0.0 | **100.0** | 0.0 | 0.0 |
|  | LEB | 0.7 | 3.2 | 5.9 | **88.7** | 0.8 | 0.7 |
|  | LFR | 3.0 | **81.1** | 4.0 | 5.9 | 3.0 | 3.0 |
| 37 | PB | 0.0 | 0.0 | 0.0 | **100.0** | 0.0 | 0.0 |
|  | LEB | 0.9 | 11.5 | 2.4 | **83.4** | 0.9 | 0.9 |
|  | LFR | 1.7 | **88.9** | 2.2 | 3.7 | 1.7 | 1.7 |
| 38 | PB | 0.0 | **100.0** | 0.0 | 0.0 | 0.0 | 0.0 |
|  | LEB | 0.1 | **99.3** | 0.1 | 0.5 | 0.1 | 0.1 |
|  | LFR | 0.3 | **98.4** | 0.3 | 0.5 | 0.3 | 0.3 |
| 39 | PB | 0.0 | 0.0 | 0.0 | **100.0** | 0.0 | 0.0 |
|  | LEB | <0.1 | <0.1 | <0.1 | **99.9** | <0.1 | <0.1 |
|  | LFR | <0.1 | <0.1 | <0.1 | **99.9** | <0.1 | <0.1 |
| 40 | PB | 0.0 | 0.0 | **50.0** | **50.0** | 0.0 | 0.0 |
|  | LEB | 0.5 | 0.6 | 16.3 | **81.3** | 0.7 | 0.6 |
|  | LFR | 1.5 | 1.8 | 23.3 | **70.4** | 1.5 | 1.5 |

| **Node** | **Analysis** | **Africa** | **South America** | **Asia** | **North America** | **Europe** | **Australia** |
| --- | --- | --- | --- | --- | --- | --- | --- |
| 41 | PB | 0.0 | 0.0 | 0.0 | **100.0** | 0.0 | 0.0 |
|  | LEB | 0.7 | 0.7 | 21.2 | **75.9** | 0.8 | 0.8 |
|  | LFR | 1.0 | 1.0 | 10.8 | **85.4** | 0.9 | 0.9 |
| 42 | PB | 0.0 | 0.0 | 0.0 | **100.0** | 0.0 | 0.0 |
|  | LEB | 0.2 | 0.2 | 1.3 | **98.0** | 0.2 | 0.2 |
|  | LFR | <0.1 | <0.1 | <0.1 | **99.9** | <0.1 | <0.1 |
| 43 | PB | 0.0 | 0.0 | **50.0** | **50.0** | 0.0 | 0.0 |
|  | LEB | 0.1 | 0.1 | 0.1 | **99.6** | 0.1 | 0.1 |
|  | LFR | 1.1 | 1.1 | 4.1 | **91.4** | 1.1 | 1.1 |
| 44 | PB | 0.0 | 0.0 | **100.0** | 0.0 | 0.0 | 0.0 |
|  | LEB | 0.1 | 0.1 | **99.2** | 0.3 | 0.1 | 0.1 |
|  | LFR | 0.6 | 0.6 | **97.0** | 0.6 | 0.6 | 0.6 |
| 45 | PB | **100.0** | 0.0 | 0.0 | 0.0 | 0.0 | 0.0 |
|  | LEB | **85.2** | 2.0 | 2.0 | 5.0 | 4.8 | 1.1 |
|  | LFR | **97.2** | <0.1 | 0.4 | 1.8 | 0.5 | <0.1 |
| 46 | PB | **33.3** | 0.0 | 0.0 | **33.3** | **33.3** | 0.0 |
|  | LEB | 27.3 | 2.8 | 2.8 | **33.6** | 30.9 | 2.5 |
|  | LFR | 41.5 | 0.7 | 0.9 | **43.5** | 12.7 | 0.7 |
| 47 | PB | 0.0 | 0.0 | 0.0 | 0.0 | **100.0** | 0.0 |
|  | LEB | 2.1 | 0.6 | 0.6 | 2.5 | **93.7** | 0.5 |
|  | LFR | 3.9 | 0.5 | 0.5 | 0.4 | **90.6** | 0.5 |
| 48 | PB | **100.0** | 0.0 | 0.0 | 0.0 | 0.0 | 0.0 |
|  | LEB | **93.2** | 2.2 | 0.7 | 0.6 | 2.8 | 0.5 |
|  | LFR | **97.7** | 1.0 | 0.3 | 0.3 | 0.3 | 0.3 |
| 49 | PB | **100.0** | 0.0 | 0.0 | 0.0 | 0.0 | 0.0 |
|  | LEB | **85.1** | 1.3 | 0.9 | 0.9 | 11.0 | 0.8 |
|  | LFR | **96.6** | 0.7 | 0.7 | 0.7 | 0.7 | 0.7 |
| 50 | PB | **100.0** | 0.0 | 0.0 | 0.0 | 0.0 | 0.0 |
|  | LEB | **97.9** | -.2 | 0.3 | 0.3 | 1.0 | 0.2 |
|  | LFR | **99.9** | <0.1 | <0.1 | <0.1 | <0.1 | <0.1 |

| **Node** | **Analysis** | **Africa** | **South America** | **Asia** | **North America** | **Europe** | **Australia** |
| --- | --- | --- | --- | --- | --- | --- | --- |
| 51 | PB | **100.0** | 0.0 | 0.0 | 0.0 | 0.0 | 0.0 |
|  | LEB | **98.1** | 0.1 | 0.8 | 0.8 | 0.1 | 0.1 |
|  | LFR | **99.9** | <0.1 | <0.1 | <0.1 | <0.1 | <0.1 |
| 52 | PB | 0.0 | **100.0** | 0.0 | 0.0 | 0.0 | 0.0 |
|  | LEB | 4.4 | **74.5** | 1.3 | 16.7 | 1.8 | 1.3 |
|  | LFR | 0.3 | **99.2** | <0.1 | 0.3 | <0.1 | <0.1 |
| 53 | PB | 0.0 | **100.0** | 0.0 | 0.0 | 0.0 | 0.0 |
|  | LEB | 0.4 | **97.6** | 0.2 | 1.2 | 0.3 | 0.2 |
|  | LFR | <0.1 | **99.9** | <0.1 | <0.1 | <0.1 | <0.1 |
| 54 | PB | **33.3** | **33.3** | 0.0 | 0.0 | **33.3** | 0.0 |
|  | LEB | **37.9** | 21.7 | 2.5 | 3.0 | 32.5 | 2.4 |
|  | LFR | **78.8** | 14.0 | 1.3 | 1.3 | 3.4 | 1.3 |
